# Sphingosine kinase 1 promotes tumor immune evasion by regulating the MTA3-PD-L1 axis

**DOI:** 10.1038/s41423-022-00911-z

**Published:** 2022-09-01

**Authors:** Poyee Lau, Guanxiong Zhang, Shuang Zhao, Long Liang, Hailun Zhang, Guowei Zhou, Mien-Chie Hung, Xiang Chen, Hong Liu

**Affiliations:** 1 Department of Dermatology, Xiangya Hospital, Central South University, Changsha, China; 2 National Engineering Research Center of Personalized Diagnostic and Therapeutic Technology, Changsha, China; 3 Hunan Key Laboratory of Skin Cancer and Psoriasis, Changsha, China; 4 Hunan Engineering Research Center of Skin Health and Disease, Changsha, China; 5 Medical Genetics & School of Life Sciences, Central South University, Changsha, Hunan 410078 China; 6 Department of Research and Development, Beijing GAP Biotechnology Co., Ltd, Beijing, 102600 China; 7 Graduate Institute of Biomedical Sciences, Research Center for Cancer Biology and Center for Molecular Medicine, China Medical University, Taichung, Taiwan China; 8 Department of Biotechnology, Asia University, Taichung, Taiwan China; 9 Department of Molecular and Cellular Oncology, the University of Texas MD Anderson Cancer Center, Houston, TX USA; 10 Xiangya Clinical Research Center for Cancer Immunotherapy, Central South University, Changsha, China

**Keywords:** Sphingosine kinase, Programmed cell death ligand 1, Programmed cell death protein 1, Melanoma, Tumor microenvironment, Immune checkpoint blockade, Skin cancer, Immune evasion

## Abstract

Immune checkpoint blockade (ICB) exhibits considerable benefits in malignancies, but its overall response rate is limited. Previous studies have shown that sphingosine kinases (SPHKs) are critical in the tumor microenvironment (TME), but their role in immunotherapy is unclear. We performed integrative analyses including bioinformatics analysis, functional study, and clinical validation to investigate the role of SPHK1 in tumor immunity. Functionally, we demonstrated that the inhibition of SPHK1 significantly suppressed tumor growth by promoting antitumor immunity in immunocompetent melanoma mouse models and tumor T-cell cocultures. A mechanistic analysis revealed that MTA3 functions as the downstream target of SPHK1 in transcriptionally regulating tumor PD-L1. Preclinically, we found that anti-PD-1 monoclonal antibody (mAb) treatment significantly rescued tumor SPHK1 overexpression or tumor MTA3 overexpression-mediated immune evasion. Significantly, we identified SPHK1 and MTA3 as biological markers for predicting the efficacy of anti-PD-1 mAb therapy in melanoma patients. Our findings revealed a novel role for SPHK1 in tumor evasion mediated by regulating the MTA3-PD-L1 axis, identified SPHK1 and MTA3 as predictors for assessing the efficacy of PD-1 mAb treatment, and provided a therapeutic possibility for the treatment of melanoma patients.

## Introduction

Malignant melanoma is a serious type of cancer originating from melanocytes. Once melanoma metastasizes, the fatality rate is alarmingly high [1, 2]. In the past decade, the number of advanced melanoma cases newly diagnosed increased by 47% annually, and this number is estimated to have increased by almost 2% in 2020 [3]. Due to research advances regarding the immunological characteristics and immunogenicity of melanoma, a series of immunotherapies have been widely applied in clinical practice [4, 5].

Sphingosine kinases (SPHKs) are essential rate-limiting enzymes with two isotypes, SPHK1 and SPHK2, that can catalyze the phosphorylation of sphingosine (SPH) to sphingosine-1-phosphate (S1P). Although the substrates of the two isotypes are identical and the amino acid sequences are highly similar, the differences in expression level and subcellular localization between SPHK1 and SPHK2 are significant [6]. SPHK1 is located in the cytoplasm in each organ, whereas SPHK2 is mainly distributed in the nucleus and specific organelles. The lines of evidence accumulated to date suggests that S1P generated by SPHK1 can be exported to the cell membrane through G protein-coupled receptors and participates in the formation of the TME. In addition, SPHK1 plays an important role in the transport of S1P via ‘inside-out’ signaling [7]. Accumulating evidence has revealed that sphingolipids are involved in normal biological processes and in cancer cells [8, 9]. Sphingosine kinases (SPHKs) are critical mediators of the “sphingolipid-rheostat” [10]. Upregulation of SPHK1 expression has been observed in many cancers, including glioma, lung, colon, and breast cancers. In addition, increased SPHK1 expression is associated with a poor survival outcomes in glioma, lung cancer, and breast cancer [11–15]. Preclinical studies have found that elevated expression of SPHK1 could result in tumor migration, invasiveness, and angiogenesis by several mechanisms, such as the SPHK1/miR-144-3p/FN1 and SPHK1/p-PAK axes [16, 17]. In addition, studies on sphingolipid-related inhibitors have made some progress. Safingol (L-threo-dihydrosphingosine), a competitive inhibitor of SPHK, is the first SPHK inhibitor investigated in clinical trials as an antitumor agent. An open-label, dose-escalating phase I trial has corroborated the pharmacokinetics and safety of safingo alone or in combination with cisplatin in patients with solid tumors [18]. Therefore, targeting SPHK1 is likely to be a key strategy for blocking the progression of cancer. Recent studies have revealed a role for SPHK1 in cancer progression [19, 20]. However, the detailed mechanisms of the involvement of SPHK1 in tumor immune escape remain unknown.

MTA3 is generally identified as an estrogen-inducible gene product that is strictly regulated by estrogen receptor α (ER-α)-positive breast cancer cell lines. Furthermore, it has been reported that MTA3 exhibits a specific ER-dependent expression pattern in the development and progression of primary breast cancers [21, 22]. In nonhormone cancers, the MTA3 mRNA and protein levels are higher in non-small-cell lung cancer tissues metastasized to the lymph node. In addition, MTA3 overexpression is associated with the pTNM stage, nodal metastasis, and poor prognosis in patients with non-small-cell lung cancer [23–25]. However, the relationship between MTA3 and PD-L1 and the role of MTA3 in the tumor microenvironment are much less clear.

By utilizing integrative analysis, SPHK1 has been associated with immune features across multiple cancer types and positively correlated with programmed cell death ligand 1 (PD-L1, also known as B7-H1/B7 Homolog1 or CD274). PD-L1 is a ligand of the B7 family. In malignant melanoma, PD-L1 expression can be induced and upregulated via innate and adaptive mechanisms. Abnormally high expression of PD-L1 on tumor cells is considered an important factor for immune tolerance and escape of immune surveillance [26]. When PD-L1 on tumor cells binds to its cognate coinhibitory receptor PD-1 expressed on tumor-infiltrating lymphocytes (TILs), the PD-L1-induced inhibitory signaling pathway can negatively regulate the activation and proliferation of T cells and promote T-cell exhaustion. The development of immune checkpoint inhibitors (ICIs) has initiated a new era for cancer therapy, and PD-1 blockade therapy is a breakthrough in this era.

Currently, a large number of drugs targeting the PD-L1/PD1 axis are approved by the Food and Drug Administration (FDA), but the therapeutic effects against solid malignancies are not satisfactory due to the complex TME and variation among individuals [26, 27]. Therefore, exploring the mechanisms of resistance to ICIs has become an important issue.

The goal of this study was to assess the role of SPHK1 in antitumor immunity, reveal a novel molecular mechanism regarding the regulation of PD-L1 via MTA3, and further investigate the clinical significance of the SPHK1-MTA3 axis in immunotherapy in melanoma.

## Materials and methods

### Cell culture

All cell lines identified by STR DNA profiling and tested for mycoplasma contamination by a MycoAlert Mycoplasma Detection Kit (Lonza#LT07-118) were proved to be negative. The human cell lines including SK-MEL-5, SK-MEL-28, and A375 were cultured in complete DMEM/F-12 (Dulbecco’s Modified Eagle Medium/Nutrient Mixture F-12) medium (Biological Industries BI#01-172-1ACS) with 10% fetal bovine serum (FBS) (BI#04-001-1ACS). The mouse melanoma cell line B16F10 was cultured in complete RPMI-1640 (Roswell Park Memorial Institute) medium (BI#01-100-1ACS) with 10% FBS. All cells were cultured in a humidified incubator with an atmosphere of 5% CO2 at 37 °C.

### Animal experiments

All animal experiments were performed following the guidelines approved by the Ethics Committee of Xiangya Hospital (Central South University, Changsha, Hunan, China). All experiments strictly adhere to the guidelines for the investigation of experimental pain in conscious animals and the minimum number of animals needed to obtain statistical significance were conducted to minimize animal suffering.

C57BL/6 wild-type female mice with specific-pathogen-free (SPF) grade were purchased at 6 weeks old from SLAC, Shanghai. All mice were housed in pathogen-free conditions at the experimental animal center, Central South University, and kept in a 12/12 h dark/light cycle with ad libitum access to food and water.

For the mouse model, 8 × 10^5^ B16F10 cells were subcutaneously injected into the right flank of anesthetized C57BL/6 mice. At 7 days after tumor implantation, when tumors had developed to be palpable, mice were randomly divided into several groups. After tumor volume and mice weight were monitored and calculated, mice in the intervention group were treated with PF543 (Selleck#S7177) (10 mg/kg or 5 mg/kg in solvent containing 5% DMSO, 5% Tween 80, 40% polyethylene glycol 300, and double-distilled H_2_O) via intraperitoneal (i.p.) injection every other day. For the control group, the solvent was administered i.p. every other day. After 4 times treatments mice were sacrificed using CO_2_ anesthetization and subsequent cervical dislocation. Tumor tissues were isolated from each mouse for further analysis. Meanwhile, anti-mouse CTLA-4 (BioXCell#BE0164) or IgG2b isotype control (BioXCell#BE0086) was co-treated with PF543 (7.5 mg/kg) or vehicle to further test whether SPHK1 inhibition by PF543 enhanced the effect of ICI therapy.

B16F10 cells infected with lentivirus overexpressing SPHK1 and MTA3 were previously prepared and administered as previously described. Anti-mouse PD-1 (BioXCell#BE0146) or IgG2a isotype control (BioXCell#BE0089) in vivo mAb treatments were conducted via i.p. (200 μg/ mouse in 100 μl Dulbecco’s phosphate-buffered saline), every three days for up to 4 times. The schematic legends of treatments were shown in the main figures.

### Flow cytometry analysis

For flow cytometry of murine samples, tumors and spleens were manually dissected, dissociated, and grinded. Mashed tissues were then filtered through 40 μM cell strainers (BD Falcon#352340). Negative compensation controls for multicolor flow cytometric analyses were established by Anti-Rat and Anti-Hamster Ig κ/Negative Control Compensation Particles Set (BD Biosciences#552845) and fluorochrome-conjugated antibody. Single-cell suspensions were then washed twice with PBS buffer. After blocking Fc receptors with TruStain FcX^TM^ anti-mouse CD16/32 antibody (Biolegend#101320) and identifying dead cells with Zombie Aqua^TM^ Fixable Viability Kit (Biolegend#423102) according to standard protocols, cells were stained with antibody against the following: APC-CD274 (Biolegend#124321), PE/ Dazzle^TM^ 594-CD274 (Biolegend#124324), BB700-CD4 (BD#566407), PerCP/Cyanine5.5-CD4 (Biolegend#100434), PE/Cyanine7-CD8a (Biolegend#100722), APC/Cyanine7-CD45 (Biolegend#103116), Brilliant Violet 421^TM^-CD279 (Biolegend#135218), Brilliant Violet 421^TM^-CD279 (Biolegend#109121), APC-CD3 (Biolegend#100236), APC-CD24 (Biolegend#102012), PE/Cyanine7-CD11b (Biolegend#101216), Brilliant Violet 421^TM^-Ly6G/Ly6C (Biolegend#108434), APC-F4/80 (Biolegend#123116), PerCP/Cyanine5.5-I-A/I-E (Biolegend#107626). For further intracellular dyeing, cells were fixed and permeabilized by Foxp3/Transcription Factor Staining Buffer Set (eBioscience#00-5523-00), then intracellular GZMB was stained using PE/Dazzle^TM^ 594-Granzyme B (Biolegend#372216), PE-FoxP3 (eBioscience^TM^#12-5773-82), PE-CD206 (Biolegend#141706) antibodies. To detect surface expression of PD-L1 on human melanoma cell lines, cells were stained by using PE-CD274 (Biolegend#329706) after blocking Fc receptors with Human TruStain FcX^TM^ (Biolegend#422302) and getting rid of dead cells with Zombie Aqua^TM^ Fixable Viability Kit (Biolegend#423102). Stained cells were detected by the DxP Athena^TM^ flow cytometry system (Cytek, USA) and the data were processed using Flow Jo Software (version 10.4).

### Cell viability assay

Tumor cells were seeded into 96-well plates at a density of 5 × 10^3^ cells per well with four duplications and pre-incubated at 37 °C and 5% CO_2_ overnight. Cells were further treated with PF543 or DMSO in gradient concentration for 24 h. The viability of tumor cells was accessed by Cell Counting Kit-8 according to the manufacturer’s instructions.

### Transfection of siRNA and lentiviral infection

Cells with the confluence of 60% were transiently transfected with duplexed si*RNAs* (2.5 nmol) by Lipofectamine^TM^ 3000 Transfection Reagent (Thermo Fisher#L3000015) according to protocols. The sequences of specific si*RNAs* were listed as follows.

**Table:** 

si*SPHK1* #1	Forward	5′-GCGUCAUGCAUCUGUUCUATT-3′
	Reverse	5′-UAGAACAGAUGCAUGACGCTT-3′
si*SPHK1* #3	Forward	5′-GUGCACCCAAACUACUUCUTT-3′
	Reverse	5′-AGAAGUAGUUUGGGUGCACTT-3′
si*MTA3* #2	Forward	5′-GUGCAACAGAAACGUCUAATT-3′
	Reverse	5′-UUAGACGUUUCUGUUGCACTT-3′
si*MTA3* #3	Forward	5′-CCAAAUAGCCCACUUACGGTT-3′
	Reverse	5′-CCGUAAGUGGGCUAUUUGGTT-3′
si*c-Myc*	Forward	5′-GUGCAGCCGUAUUUCUACUTT-3′
	Reverse	5′-AGUAGAAAUACGGCUGCACTT-3′

To establish stable overexpression cell lines, HEK293FT cells were cotransfected with packaging plasmids (pMD2G and pspAX2) and pLVX-Puro-Flag-SPHK1, pLVX-Puro-Flag-MTA3, pLVX-Puro-EV, pCDH-CMV-MCS-EF1-copGFP-T2A-Puro-SPHK1, pCDH-CMV-MCS-EF1-copGFP-T2A-Puro-MTA3, or pCDH-CMV-MCS-EF1-copGFP-T2A-Puro-EV plasmids using Lipofectamine 3000 Transfection Reagent. 48 h after transfection, virus-containing supernatants were collected separately and cells were cultured with the virus supernatants for 48 h. Then infected cells were selected by puromycin (1 μg/ml) for 5 days. The efficiency of transfection was then confirmed on transcription and translation levels.

RNA oligos were synthesized and purchased from GenePharma. Packaging plasmids (pMD2G and pspAX2) were purchased from Thermo Scientific. Plasmids pLVX-Puro-Flag-SPHK1, pLVX-Puro-Flag-MTA3, pLVX-Puro-EV, pCDH-CMV-MCS-EF1-copGFP-T2A-Puro-SPHK1, pCDH-CMV-MCS-EF1-copGFP-T2A-Puro-MTA3, and pCDH-CMV-MCS-EF1-copGFP-T2A-Puro-EV were purchased from Vigene Biosciences.

### RNA extraction and quantitative real-time PCR assay

Total RNAs were extracted from cells using MagZol^TM^ Reagent (Magen#R4801-01) and cDNA was synthesized using HiScript II^®^ Q RT SuperMix for qPCR (+gDNA wiper) (Vazyme#R223) according to manufacturer’s protocol. qRT-PCR was performed using UltraSYBR Mixture (CWBIO#CW0957) on QuantStudio-3 Real-Time PCR systems (Thermo Fisher Scientific). The data were analyzed using the fold change of gene expression with the formula 2^−ΔΔCT^ method.

All sequences of PCR primers used in this study are as follows:

**Table:** 

Human-GAPDH	Forward	5′-GCCCAATACGACCAAATCC-3′
	Reverse	5′-CTCTGCTCCTCCTGTTCGAC-3′
Human-CD274	Forward	5′-CCAGTCTCTGAACATGAA-3′
	Reverse	5′-ATTGGTGGTGGTGGTCTTAC-3′
Human-SPHK1	Forward	5′-TGCTGTCACCCATGAACC-3′
	Reverse	5′-CCCAGACGCCGATACTTC-3′
Human-MTA3	Forward	5′-AGCCCACTTACGGATCGACAGA-3′
	Reverse	5′-CAAACTAGGCTGCCTCACAGAAC-3′
Human-c-Myc	Forward	5′-CTACCAGCAGCAGCAGCAGAG-3′
	Reverse	5′-GGTGTGACCGCAACGTAGGAG-3′
Human-GATA3	Forward	5′-ACCACAACCACACTCTGGAGGA-3′
	Reverse	5′-TCGGTTTCTGGTCTGGATGCCT-3′
Human-MYBL2	Forward	5′-TCAGAAGTACTCCATGGACAAC-3′
	Reverse	5′-GTCCTCGATGATGAGTTCGAT-3′
Human-SMAD1	Forward	5′-TTGGCACAGTCTGTGAACCATGG-3′
	Reverse	5′-GTAACATCCTGGCGGTGGTATTC-3′
Human-STAT5A	Forward	5′-GCTGTTGCCCACGTTTC-3′
	Reverse	5′-CTGTCCACCCACCATATCC-3′
Human-NFATC1	Forward	5′-GCCATCCTCTCCAACACC-3′
	Reverse	5′-CCGATGTCCGTCTCTCCT-3′
Human-UBTF	Forward	5′-CCGAATGTGGAACGACCTGTCT-3′
	Reverse	5′-GATCTCCTCAGCTCTTTTGGGG-3′
Human-TFAP4	Forward	5′-GCAGCCATTCTCCAGCA-3′
	Reverse	5′-TCGTCCTCCCAGATGTCC-3′
Human-E2F1	Forward	5′-GGACCTGGAAACTGACCATCAG-3′
	Reverse	5′-CAGTGAGGTCTCATAGCGTGAC-3′
Mouse-GAPDH	Forward	5′-GGTTGTCTCCTGCGACTTCA-3′
	Reverse	5′-TGGTCCAGGGTTTCTTACTCC-3′
Mouse-CD274	Forward	5′-GCTCCAAAGGACTTGTACGTG-3′
	Reverse	5′-TGATCTGAAGGGCAGCATTTC-3′

### Western blot

Cells were cultured and lysed at 4 °C for 30 min in RIPA buffer (Beyotime#P0013C) supplemented with protease inhibitors (Selleck#S3025) and phosphatase inhibitors (Selleck#S3620) after phosphate buffer saline (PBS) washing. A Bicinchoninic Acid Protein Assay Kit (CWBIO#CW1104S) was used to measure the protein concentration. Equal quantities of protein were separated using SDS-polyacrylamide gel electrophoresis (SDS-PAGE) on a 10% gel and transferred on PVDF membranes (Merck Millipore Ltd.). After blocking in 5% nonfat milk dissolved by Tris Buffered saline Tween (TBST) for 1 h, immunoblotting was performed with primary antibodies at 4 °C overnight. Then membranes were incubated with horseradish peroxidase-conjugated secondary antibodies (ABclonal) at room temperature for 1 h after being washed by TBST. Immunoreactions were visualized using a NcmECL Ultra kit (NCM Biotech#P10300). Image acquisition and quantitation of intensity were performed by Odyssey^®^ Fc Imaging System (LI-COR Biosciences, Lincoln, NE, USA).

### T cell culture and T cell-mediated killing assay

To obtain activated T cells, human peripheral blood mononuclear cells (PBMC; LTS1077, Yanjin Biological) were cultured in AIIM V^TM^ Medium CTS^TM^ (Gibco#A3021002) containing 10% FBS (Gibco#10099141 C), ImmunoCult^TM^ Human CD3/CD28/CD2 T Cell Activator (STEMCELL Technologies#10970) and Recombinant Human IL-2 Protein (R&D#202-IL-050, 20000 IU/μg) for one week according to the manufacturer’s instructions. The experiments were performed with CD3 Monoclonal Antibody (eBioscience Thermo Scientific#16-0037-81, 100 ng/mL) and IL-2 (1000 IU/mL). Tumor cells were seeded and allowed to adhere to the 96-well plates overnight and then incubated for 48 h with activated T cells in the presence or absence of PF543. The effector-to-target (E-T) ratio between activated cells and tumor cells was utilized in terms of the purpose of each experiment. T cells and the debris of cells were removed and washed by PBS, and then living tumor cells were quantitatively analyzed by a spectrometer (Beckman Coulter DU-800) at OD (450 nm) followed by Cell Counting Kit-8 (Bimake#B34302) staining.

### ChIP-qPCR assay

The ChIP assay was conducted using SimpleChIP^®^ Enzymatic Chromatin IP Kit (Magnetic Beads) (Cell Signaling Technology#9003 S) following the manufacturer’s instructions. Briefly, for each immunoprecipitation, cells were cultured to approximately 1 × 10^7^ and cross-linked by 1% diluted formaldehyde (Sigma-Aldrich#252549). Cells were then harvested and digested chromatin with micrococcal nuclease. Next, sonicate up to lysate with several pulses to break nuclear membrane. DNA fragment was checked to a length of approximately 100–900 bp. Chromatin was immunoprecipitated by negative control Normal Rabbit IgG (Cell Signaling Technology#2729) or Flag antibody (1:100, Cell Signaling Technology#14793) at 4 °C with rotation overnight. After eluting chromatin from Antibody/Protein G Magnetic beads and reversing cross-links, the purified DNAs were further quantified by PCR. The sequences of PCR primers are listed below:

**Table:** 

c-Myc	Forward#1	5′-GGAGGCTATTCTGCCCATTT-3′	536–556 bp
	Reverse#1	5′-AAGTGGACTTCGGTGCTTAC-3′	653–673 bp
	Forward#2	5′-AGGGATCGCGCTGAGTATAA-3′	216–236 bp
	Reverse#2	5′-TCTGCCTCTCGCTGGAATTA-3′	272–292 bp
	Forward#3	5′-AAACCAGGTAAGCACCGAAG-3′	646–666 bp
	Reverse#3	5′-TGTCAATAGCGCAGGAATGG-3′	744–764 bp

### Multiplex immunohistochemical assay and analysis

Sections (4–6 μm) from formalin-fixed and paraffin-embedded melanoma samples were deparaffinized and rehydrated through a graded series of ethanol. All slides were fixed in 10% neutral buffered formalin for 30 min. Multiplex staining was performed by the same primary antibodies as immunohistochemistry (IHC) analysis with subsequent antibody detections using Opal Polymer HRP Ms + Rb reagents, and corresponding fluorophores as well as spectral DAPI nuclear counterstain were applied following the manufacturer’s instructions of Opal 7-color manual IHC kit (NEL811001KT, PerkinElmer). The slides were scanned and captured using the Vectra^®^ Polaris^™^ Automated Quantitative Pathology Imaging System (PerkinElmer) with fluorescent imaging mode. Each multispectral image cube was then separated into individual components by the spectral library to identify all target markers in a single image using inForm^®^ advanced image analysis software (version 2.5.0). All multispectral images of tissue sections with spectral unmixing and segmentation were further subjected to create a phenotyping algorithm based on machine active learning. Phenotype quantifications and analyses were conducted blinded to the samples and outcomes.

**Table:** 

Antigen	Primary antibody		TSA dyes
	Dilution	Company/Catalog number	
**Mouse**			
CD8α	1:20000	Αbcam/ ab209775	690
PD-L1	1:10000	Proteintech/ 622848-1-Ig	620
**Human**			
SPHK1	1:500	Abcam/ ab71700	570
MTA3	1:500	Proteintech/ 14682-1-AP	520
c-Myc	1:500	Santa Cruz/ sc-40	620
PD-L1	1:10000	CST/ 13684	690
GZMB	1:1000	Abcam/ ab4059	570
CD8	1:20000	Proteintech/ 66868-1-Ig	520
CD45	1/2000	Abcam/ ab208022	690
CD3	1/1000	Abcam/ ab16669	620

### Clinical tissue samples

Metastatic melanoma tissue microarrays were purchased from Avilabio. Paraffin sections of melanoma patients treated with PD-1 mAb (Toripalimab) were obtained from Xiangya Hospital, Central South University. All tissue samples were collected in compliance with the informed consent policy. The study protocol was approved by the respective institutions’ institutional review boards or ethics committees. Clinical information is summarized in Supplementary Table 9.

### Bioinformatics analysis and RNA sequencing

Gene transcriptome data of 33 cancer types including 10205 carcinoma samples and immune cell populations based on Fragments Per Kilobase per Million (FPKM) methods were downloaded from The Cancer Genome Atlas (TCGA) database (http://gdac.broadinstitute.org/) [28]. Differential expression analysis for TCGA was performed by limma (v1.4.5) with |Log_2_(fold change)| >0.58 and adjusted *p* < 0.05 based on the Benjamini-Hochberg (BH) method. The score of immune cell types was calculated with gene set variation analysis (GSVA). Gene list of each immune cell type was derived from Charoentong et al. [29].

The total infiltration score of immune cells was calculated by Immune Cell Abundance Identifier (ImmuCellAI) [30, 31]. Spearman’s rank correlation coefficient (*Rs*) was calculated between SPHK1 expression and immune cell profiles or inhibitory immune checkpoint expression. Statistical significance was considered with |*Rs* | >0.3 and *p* < 0.05.

Processed data for papillary thyroid cancer cells with SHPK1 overexpression and control were downloaded from the GEO database (GSE87307 [17]). GO enrichment analyses were performed with significantly upregulated genes by hypergeometric test, and KEGG enrichment analyses were performed by the GSEA method.

For correlation analysis between SPHK1 and candidate transcription factors, the gene expression data of 52 melanoma cells were downloaded from the GDSC database (https://www.cancerrxgene.org/). The dataset for melanoma patients receiving anti-PD-1 immunotherapy (GSE78220 [32]) was downloaded from the GEO database (https://www.ncbi.nlm.nih.gov/geo/).

Survival analyses were performed using the survival and survminer packages in R (http://www.r-project.org, version 4.0.3). Log-rank tests were used to compare overall survival between patients in different groups. Treatment data of melanoma samples receiving with or without immunotherapy were obtained from the TCGA database. Samples were subdivided into two groups based on median expression or the specific quantile cutoff.

RNA-sequencing was performed by DNA nanoball-seq (DNBSEQ) genetic sequencer to generate pair-end 150 bases reads in the way of combinatorial Probe-Anchor Synthesis. Detailed procedures about cDNA library establishment, library quality control, and sequencing were implemented according to the technical instructions of BGI Life Science Research Institution (https://www.genomics.cn/). The original sequencing data was filtered with FastQC (version 0.11.9) to obtain clean reads. The clean reads were then mapped to the reference genome using HISATS (version 2.0.4). RSeQC (version 3.0.1) was applied to further align the clean reads to the reference gene set, then the expression level of the gene was calculated by RSEM (version 1.2.12). Differential expression analysis was performed by DESeq2 (version 1.4.5) with a Q value less than 0.05.

### Statistical analysis

For the tumor growth data analysis, no experiments displaying obvious bias in starting tumor volume as the equality of mice randomization was examined by the statistical analysis. The overall difference at each data collection time point was tested by one-way ANOVA. For the comparisons between specific groups, statistical significance was determined by one-way fixed-effect model ANOVA followed by Tukey’s multiple comparisons test. To evaluate the treatment efficacies, a linear mixed effect model (SPSS, version 24) was conducted by setting the individual mouse subject as a random effect, and treatments, day, weight as well as the interactions of treatments and days as fixed effects. Model adequacy diagnostics were performed to access the assumption of normality, linearity, and homogeneity of variances of residuals generated by the model. Model fitting results were presented only when all assumptions are not violated.

For the association analyses between PD-L1, SPHK1, and MTA3 expression levels, Chi-square tests were used to examine the statistical significance in the 2 × 2 tables. If all the expected numbers were greater than 5. If an expected number was less than 5, Fisher’s exact tests were applied to assess the significance. Two-sided unpaired Student’s *t*-tests were used to calculate the statistical differences between the two groups. Differences between multiple groups were determined by one-way ANOVA and Dunnett’s multiple comparison test. *P* values less than 0.05 were considered statistically significant and represented as *p < 0.05, **p < 0.01, ***p < 0.001, and ****p < 0.0001. All quantitative data were obtained from at least three independent experiments. Experimental data were analyzed and visualized using GraphPad Prism software (GraphPad Software, lnc., version 8.0.1) and R (version 4.0.3).

## Results

### Dysregulation of SPHK1 and associations with tumor immune characteristics

**Fig. 1:**
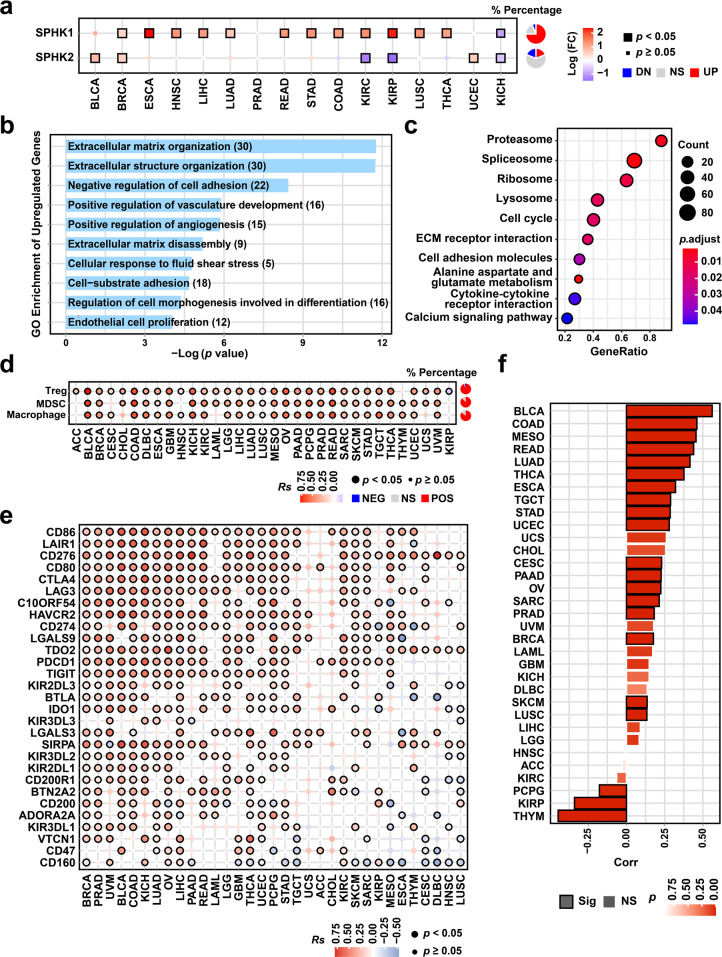
Asscociations between SPHK1 and tumor-infiltrating lymphocytes and inhibitory biomarkers. **a** Differential expression of SPHK1 and SPHK2 across 16 cancer types compared with normal samples (identified by limma, |Log_2_(fold change)| >0.58, BH-adjusted *p* < 0.05). Pie charts in the right panel represent the percentage of cancer types with significant upregulation (red; UP), downregulation (blue; DN), and non-significant alteration (gray; NS). GO (**b**) and KEGG (**c**) functional analysis of differential expressed genes between papillary thyroid cancer cells with SHPK1 overexpression and control (GSE87307). GO enrichment was performed with upregulated genes by hypergeometric test, and KEGG enrichment was performed by the GSEA method. **d** Correlation between SPHK1 and the relative abundance of suppressive immune cell types including regulatory T cells (Tregs), myeloid-derived suppressor cells (MDSCs), and tumor-associated macrophages (TAMs). Pie charts in the right panel represent the percentage of cancer types with significantly positive (red; POS), negative (blue; NEG), and non-significant correlation (gray; NS). **e** Correlation between SPHK1 and inhibitory checkpoint gene expression in 33 cancer types. **f** Correlation between SPHK1 and CD274 across 33 cancer types from the TCGA database

The overexpression of SPHK1 contributes to tumor progression and development, but the role of SPHK1 in antitumor immunity is unclear [33, 34]. We conducted gene set variation analyses to calculate the relative expression levels of SPHKs by comparing tumor and normal tissues across sixteen cancer types using data from The Cancer Genome Atlas (TCGA) [35]. We observed significant differential expression of SPHK1 in multiple cancers compared with that of SPHK2, which suggested the global ubiquity of SPHK1 dysregulation in cancers (Fig. 1a). Based on these data, we found that SPHK1 was overexpressed in thirteen cancer types, including breast invasive carcinoma (BRCA, Log(FC) = 0.52, *p* < 0.001), head and neck squamous cell carcinoma (HNSC; Log(FC) = 1.30, *p* < 0.001), liver hepatocellular carcinoma (LIHC; Log(FC) = 1.23, *p* = 0.002), lung squamous cell carcinoma (LUSC; Log(FC) = 1.32, *p* < 0.001), and thyroid carcinoma (THCA; Log(FC) = 1.17, *p* < 0.001). The mRNA profiles of the papillary thyroid cancer cell line TPC1 with SPHK1 overexpression were further obtained by sequencing to assess the roles of SPHK1 [17]. Moreover, we analyzed the correlations between SPHK1 expression and cancer hallmarks collected from the Molecular Signatures Database (MSigDB), which is a well-defined oncogenic axis in multiple cancers [35]. By Gene Oncology (GO) enrichment analysis, we found that the upregulated genes induced by SPHK1 were significantly enriched in GO terms including cell cycle and extracellular matrix (ECM)-related pathways (Fig. 1b, c). In addition, SPHK1 gene expression was correlated with epithelial mesenchymal transition, the interferon (IFN)-α/γ response, and the transforming growth factor (TGF)-α/β signaling pathway, which indicated the potential effects of SPHK1 on the TME (Supplementary Fig. 1a-f). Furthermore, we systematically examined the potential functions of SPHK1 in antitumor immunity and found significant associations between SPHK1 expression levels and the abundance of suppressive tumor-infiltrating immune cell subsets, including regulatory T cells (Tregs; median *Rs* = 0.36), myeloid-derived suppressor cells (MDSCs; median *Rs* = 0.37), and tumor-associated macrophages (TAMs; median *Rs* = 0.32) across 33 cancer types (Fig. 1d). In addition, a correlation analysis was performed between SPHK1 and immune checkpoint molecule expression levels in 33 human cancers. Strong correlations were detected between SPHK1 and a majority of inhibitory immune checkpoint molecules (Fig. 1e). For example, PD-1 (encoded by PDCD1) and PD-L1 (encoded by CD274), a well-defined immunosuppressive axis in the TME [36–38], were found to be correlated with SPHK1 expression in most cancer types, including skin cutaneous melanoma (SKCM; Fig. 1f, g). These results suggest that SPHK1 possesses immunomodulatory potential, as reflected by the changes in tumor-infiltrating immune cells and the generally increased levels of inhibitory checkpoints in the TME.

### The inhibition of SPHK1 suppresses tumor growth by reversing PD-L1-mediated tumor immune escape and leads to activation of T lymphocytes in immunocompetent mouse models and melanoma cell lines

**Fig. 2:**
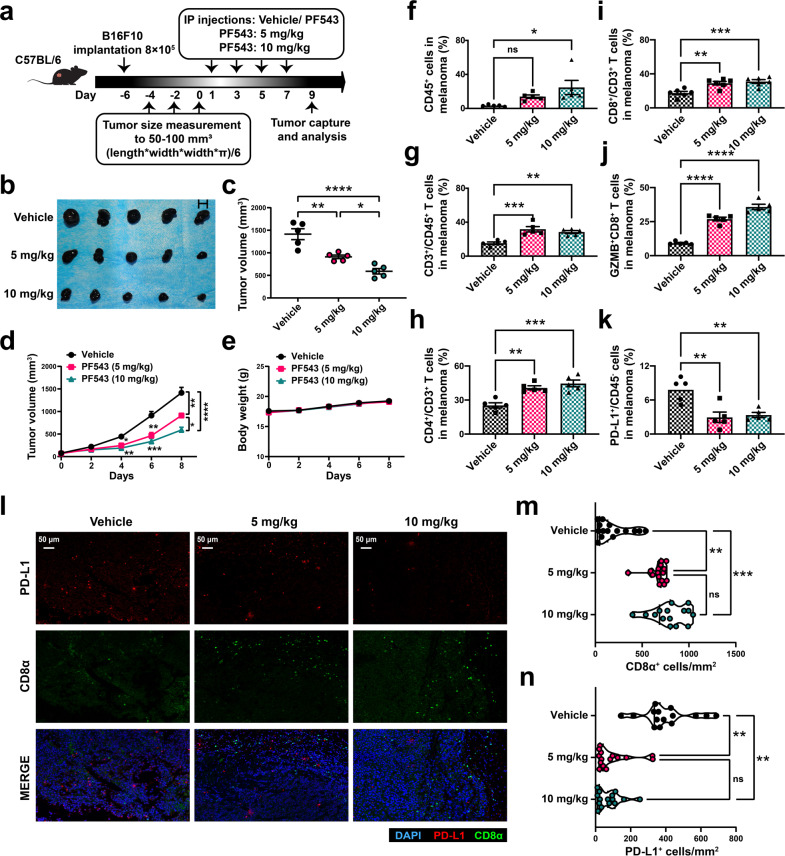
Inhibition of SPHK1 suppresses tumor growth by promotion of anti-tumor immunity in melanoma immunocompetent mouse models. **a** Schematics of the treatment plan using a C57BL/6 female mouse model, established by subcutaneously injecting B16F10. Mice were then either treated with high-dose PF543 (10 mg/kg), low-dose PF543 (5 mg/kg), or solvent (vehicle). **b** Image of B16F10 tumors, as captured on the ninth day. **c** Summary tumor volume data harvested on the ninth day. Plots of mice tumor volumes (**d**) and body weight (**e**) measured every other day. See also Supplementary Tables 1-2. Flow cytometric analyses of CD45^+^ cells (**f**), CD3^+^ in CD45^+^ cells (**g**), CD4^+^ or CD8^+^ in CD3^+^ cells (**h**, **i**), GZMB^+^CD8^+^ TILs (**j**), and PD-L1 on CD45^-^ subsets (**k**) from B16F10 tumor samples. See also Supplementary Fig. 2a–e. **l** Representative images of trichrome immunohistochemical staining of DAPI, CD8α, and PD-L1 of B16F10 tumor-bearing mice treated with vehicle, PF543 (5 mg/kg or 10 mg/kg). Scale bars, 50 μm. Violin plots indicating densities of CD8α^+^ (**m**) and PD-L1^+^ (**n**) cells/mm^2^ in IHC sections of B16F10 allografts. 5 mice per cohort. Data represent mean ± SEM. ns non-significant, *p* > 0.05, *p < 0.05, **p < 0.01, and ***p < 0.001, as determined by one-way ANOVA and Tukey’s multiple comparisons test (**c**, **f**–**k**, **m**, **n**)

To investigate the potential effects of SPHK1 on malignant melanoma, PF543, a specific SPHK1 inhibitor, was utilized in immunocompetent melanoma mouse models. Specifically, we inoculated wild-type (WT) B16F10 mouse melanoma cells into C57BL/6 mice for an in vivo study (Fig. 2a). The tumor size was significantly decreased in a dose-dependent manner in the group administered PF543 compared with the control group (Fig. 2b–d and Supplementary Tables 1, 2). Moreover, we found no significant fluctuation in body weight during PF543 treatments, which suggested that PF543 had limited dose toxicity in mice bearing melanoma (Fig. 2e). Flow cytometric analyses showed that PF543 treatment significantly induced tumor-infiltrating CD4^+^ T cells and CD8^+^ T cells in the TME (Fig. 2f–i and Supplementary Fig. 2a–c). Of note, the frequency of granzyme B (GZMB)-producing CD8^+^ cytotoxic T lymphocytes (CTLs) was increased substantially in the PF543 group versus the vehicle control group (Fig. 2j and Supplementary Fig. 2d). Because GZMB is an identified marker with the strongest cytotoxic capacity toward target cells [39–41], these results indicate that PF543 treatment may increase the tumor-specific CD8^+^ CTL population in the TME. Furthermore, decreased tumor PD-L1 expression was observed in the PF543 group compared with the control group, and the PD-1 expression on CD45^+^ cells was slightly decreased after the PF543 treatment (Fig. 2k and Supplementary Fig. 2e–g). Similarly, multiplex immunohistochemical (IHC) staining based on tyramide signal amplification (TSA) and quantitative analyses showed a greater density of CD8^+^ T cells and a reduced number of PD-L1^+^ cells in the PF543 group compared with the vehicle control group (Fig. 2l–n). Consistent with previous analyses, we found that inhibition of SPHK1 activity significantly diminished the levels of CD4^+^CD25^+^FoxP3^+^ regulatory T cells (Tregs) and Gr-1^+^CD11b^+^ myeloid-derived suppressor cells (MDSCs) in melanoma (Supplementary Fig. 2h–k). Taken together, our results indicated that SPHK1 might play a pivotal role in the TME and that an SPHK1 inhibitor has the potential to facilitate an antitumor response by downregulating PD-L1 on melanoma cells and thereby reversing the dysfunctional status of tumor-infiltrating CTLs.

To further decipher the efficacy of PF543 plus ICI, we utilized a combination strategy involving cotreatment with PF543 and CTLA-4 mAb because the blockade of CTLA-4 is involved in promoting T-cell activation and elevating dendritic cell (DC) activity via the Tregs (Supplementary Fig. 3a) [42–46]. The results showed that CTLA-4 mAb treatment significantly decreased the tumor volume compared with the IgG2b group (Supplementary Fig. 3b–e and Supplementary Tables 3, 4). Notably, cotreatment with PF543 and CTLA-4 mAb further restrained tumor growth compared with that obtained with PF543 or CTLA-4 mAb treatment alone. Using flow cytometry analyses, we observed that the CTLA-4 mAb led to a marked reduction in CD4^+^CD25^+^FoxP3^+^ Tregs in melanoma, consistent with early studies (Supplementary Fig. 3i, m). More importantly, the activity (GZMB^+^) of infiltrated CD8^+^ T cells was significantly restored in the combination group compared with that in the PF543 or CTLA-4 mAb monotherapy group (Supplementary Fig. 3f–h, m). Strikingly, compared with the vehicle control, PF543 treatment substantially reduced CD11b^+^F4/80^+^CD206^+^MHC class II^-^ tumor-associated macrophages (TAMs) and Gr-1^+^CD11b^+^ MDSCs, but did not contribute to the tumor infiltration of CD11b^+^F4/80^+^MHC class II^+^CD206^-^ cell subsets (Supplementary Fig. 3j–m). Together, the results indicate that the effect of PF543 on tumor-infiltrating CTL activation and the profound association of PF543 and attenuated immunosuppressive cells indicate that PF543 likely contributes to augmented CTLA-4 mAb efficacy.

To validate the effects of SPHK1 on the regulation of PD-L1 expression by tumor cells, melanoma cell lines were treated with PF543. Consistent with our hypothesis, we found that inhibition of SPHK1 significantly suppressed both the mRNA and protein levels of PD-L1. (Supplementary Fig. 4a–c and Supplementary Fig. 6a–c). In addition, flow cytometric analysis showed that IFN-γ-induced surface expression of PD-L1 decreased in a dose-dependent manner following PF543 treatment in melanoma cell lines (Supplementary Fig. 6d, e). To further assess the functional role of SPHK1-mediated regulation of tumor PD-L1, a human peripheral blood mononuclear cell (PBMC)-mediated tumor cell killing assay was conducted. Compared with control cells, cells treated with PF543 tended to exhibit decreased resistance to CTL-mediated target cell killing (Supplementary Fig. 6f). Altogether, these findings support the notion that the inhibition of SPHK1 by PF543 suppresses tumor cell growth by decreasing PD-L1 expression and subsequently enhancing CTL activity.

### MTA3 functions as a downstream target of SPHK1 to regulate PD-L1 in melanoma

**Fig. 3:**
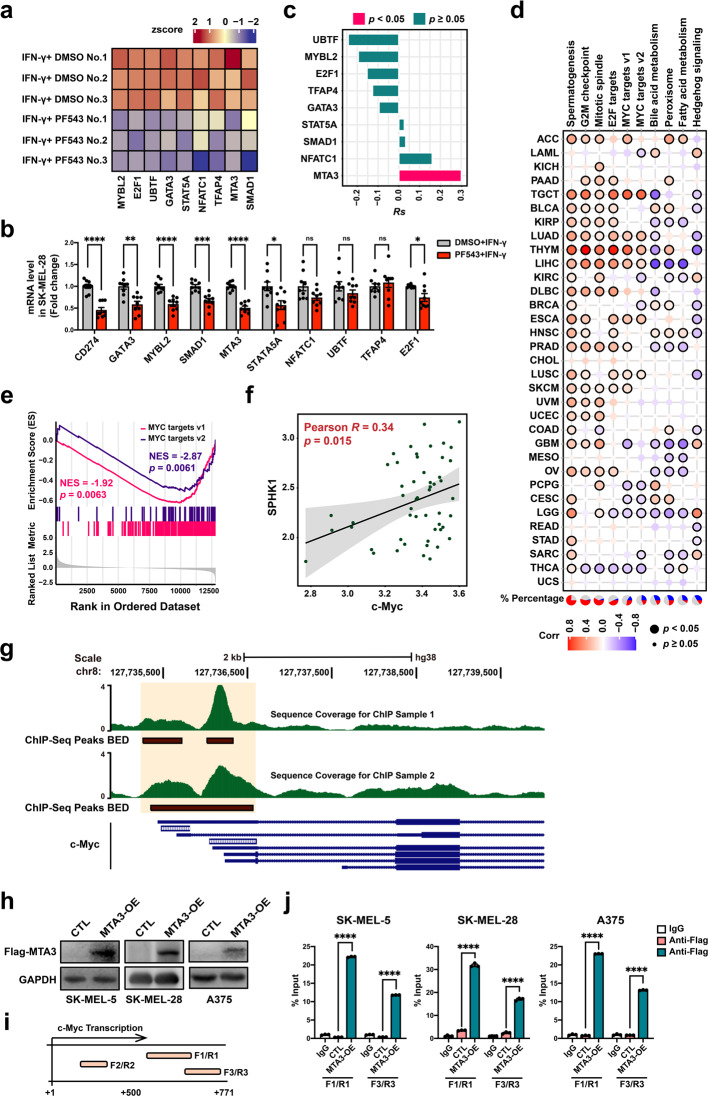
SPHK1 transcriptionally regulates MTA3 and c-Myc in melanoma cells. **a** Heat map of RNA-seq expression z-scores computed the candidate genes in SK-MEL-28 melanoma cells treated with PF543 (25 μM) and IFN-γ (200 ng/mL) for 24 h. See also Supplementary Table 5. **b** RT-PCR of CD274 and candidate transcription factors mRNA level was performed from the same sample as RNA-seq (*n* = 9). Data represent mean ± SEM. ns non-significant, *p* > 0.05, *p < 0.05, **p < 0.01, ***p < 0.001, and ****p < 0.0001. *P* values were calculated using unpaired two-sided Student’s *t*-test. **c** SPHK1 was positively correlated with MTA3 by using the calculation of Pearson’s correlation coefficient and subsequent significance test in 52 melanoma cell lines from the GDSC database. **d** Spearman’s correlation between MTA3 expression and MSigDB hallmark pathways across 33 cancer types. Pie charts in the left panel represent the percentage of cancer types with positive (red, *Rs* > 0.2, *p* < 0.05), negative (blue, *Rs* < −0.2, *p* < 0.05), and non-significant correlation (gray). **e** Enrichment plot of MYC target gene sets based on RNA sequencing. **f** Scatterplot for linear-regression of the association between c-Myc and SPHK1, as determined by Pearson’s correlation coefficient and significance test. The solid line corresponds to the regression estimate, and the corresponding 95% CIs, indicated by grey shading. **g** Overlaid images of MTA3 ChIP-seq enrichment from K562 cell line for two replicates. ChIP-seq tracks are obtained from published data. **h** SK-MEL-28 melanoma cells were transfected with MTA3 or negative control (CTL) vector. MTA3 expression was analyzed by western blot. **i**, **j** Locations of ChIP-qPCR primers at the c-Myc promoter, transcription start site is designated as nucleotide +1. F/R, Forward/Reverse (**i**). Input % of c-Myc DNA by IgG or Flag antibody were determined by ChIP-qPCR in melanoma cell lines (*n* = 3). **j** Data represent mean ± SEM. ns non-significant, *p* > 0.05, *p < 0.05, **p < 0.01, ***p < 0.001, and ****p < 0.0001. *P* values were calculated using unpaired two-sided Student’s *t*-test

We next sought to explore the downstream signaling pathway underlying the SPHK1-induced regulation of tumor PD-L1 expression levels in melanoma. RNA-sequencing (RNA-seq) analysis was performed with an IFN-γ-stimulated SK-MEL-28 melanoma cell line treated with PF543 or dimethyl sulfoxide (DMSO). Among all the downregulated genes, nine candidate transcription factors (TFs) were identified based on the fold changes in differentially expressed genes (Fig. 3a and Supplementary Table 5). To further assess the robustness of the bioinformatic analysis, isolated RNA samples were utilized for qRT-PCR quantification (Fig. 3b). Correlation analyses were performed between SPHK1 and the aforementioned TFs using data from the Genomics of Drug Sensitivity in Cancer (GDSC) database. The results showed a significantly positive correlation between SPHK1 and MTA3 (Fig. 3c). Furthermore, the MTA3 expression levels were correlated with cancer hallmarks, including the G2/M checkpoint, E2F1 targets, and MYC targets (Fig. 3d). Among the downstream genes, genes with downregulated expression were significantly enriched in c-Myc target pathways (Fig. 3e), and this finding was further validated by correlation analysis based on the GDSC database (Fig. 3f). We further explored the correlation between MTA3 and c-Myc by retrieving genome-wide chromatin immunoprecipitation (ChIP) sequencing data from the GEO repository [47]. We found that MTA3 peaks localize close to the c-Myc promoter region in the K562 chronic myelogenous leukemia cell line, implying that it might regulate c-Myc transcriptionally (Fig. 3g). Next, we performed ChIP quantitative PCR assays of melanoma cell lines to validate c-Myc as the target gene of MTA3, and MTA3 was found to directly bind at the region of the c-Myc promoter (Fig. 3h–j).

**Fig. 4:**
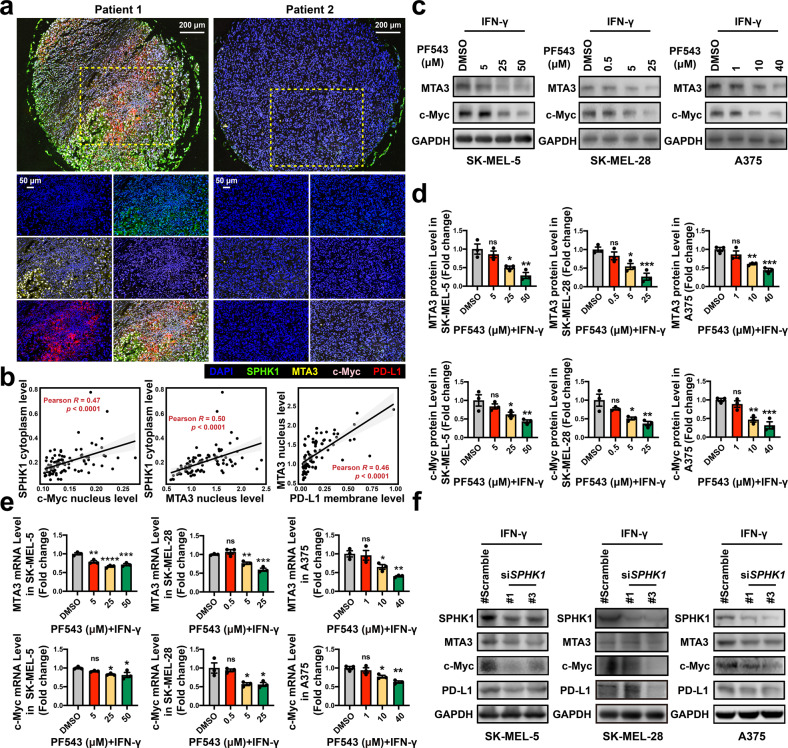
SPHK1 and MTA3 are positively correlated with PD-L1 expression in melanoma patient samples. **a** Representative images of tetrachromatic immunohistochemical staining of DAPI, SPHK1, PD-L1, and MTA3 expression in a metastatic melanoma tissue array. Scale bars, 200/50 μm in insets. **b** Quantification and correlation analysis of SPHK1, PD-L1, and MTA3 based on Log_2_-transformed fluorescent intensities of multiplex IHC staining. See also Supplementary Table 6. MTA3 or c-Myc expression was analyzed by western blot including images (**c**) and quantification (**d**), or RT-PCR (**e**; *n* = 3) in melanoma cell lines treated with PF543 or DMSO. The plot (**d**) was generated from three independent experiments and showed as mean ± SEM. **f** Melanoma cells transfected with si*SPHK1* or scrambled negative control si*RNA* were visualized by western blot. See also Supplementary Fig. 7. Data represent mean ± SEM. ns non-significant, *p* > 0.05, *p < 0.05, **p < 0.01, ***p < 0.001, and ****p < 0.0001, as determined by Dunnett’s multiple comparisons test (**d**, **e**)

To confirm our findings in patient samples, we detected protein expression levels in tumor tissues from a cohort of 90 melanoma patients by multiplex IHC staining. The results showed that the SPHK1 expression levels were positively correlated with MTA3 (*p* < 0.0001) and c-Myc (*p* = 0.020). In addition, the MTA3 levels were positively correlated with the PD-L1 levels (*p* < 0.0001) (Fig. 4a, b and Supplementary Table 6).

Consistent with our observations, the suppression of SPHK1 in melanoma cell lines in vitro, either by treatment with PF543 (Fig. 4c–e) or with an SPHK1-targeting short interfering RNA (si*RNA*; Fig. 4f and Supplementary Fig. 7a, b), produced reductions in the MTA3 and c-Myc expression levels. Together, these results suggest that MTA3 is the downstream target of SPHK1 that regulates PD-L1.

Next, we investigated the mechanism underlying the MTA3-mediated regulation of PD-L1. Our results showed that the knockdown of MTA3 resulted in significantly decreased levels of IFN-γ-induced PD-L1 and c-Myc in melanoma cell lines in vitro (Supplementary Fig. 8a–c). To further confirm these mechanistic observations, we subsequently knocked down c-Myc in MTA3-overexpression (OE) cell lines or control (empty vector, EV) cells. Consistently, MTA3 overexpression enhanced the inducible PD-L1 levels compared with the control expression levels (Supplementary Fig. 8d, e). Of note, there was a statistically significant difference in the upregulation of PD-L1 was detected after the knocking down of c-Myc in the MTA3-OE group versus the knockdown of c-Myc in the MTA3-EV group, indicating that MTA3 positively regulates PD-L1 in a c-Myc-independent manner. Based on the aforementioned evidence, we inferred that SPHK1 positively regulates PD-L1 expression through MTA3. To test this hypothesis, we established melanoma cell lines stably overexpressing Flag-SPHK1 driven by lentiviral vectors and subsequently knocked down MTA3. The results showed that SPHK1-induced PD-L1 protein expression could be rescued by MTA3 suppression (Supplementary Fig. 8f and Supplementary Fig. 9). Thus, we provide evidence showing that SPHK1 positively regulates PD-L1 expression in an MTA3-dependent manner.

### PD-L1 expression induced by SPHK1 and MTA3 is related to the efficacy of an anti-PD-1 mAb in immunocompetent mouse models

**Fig. 5:**
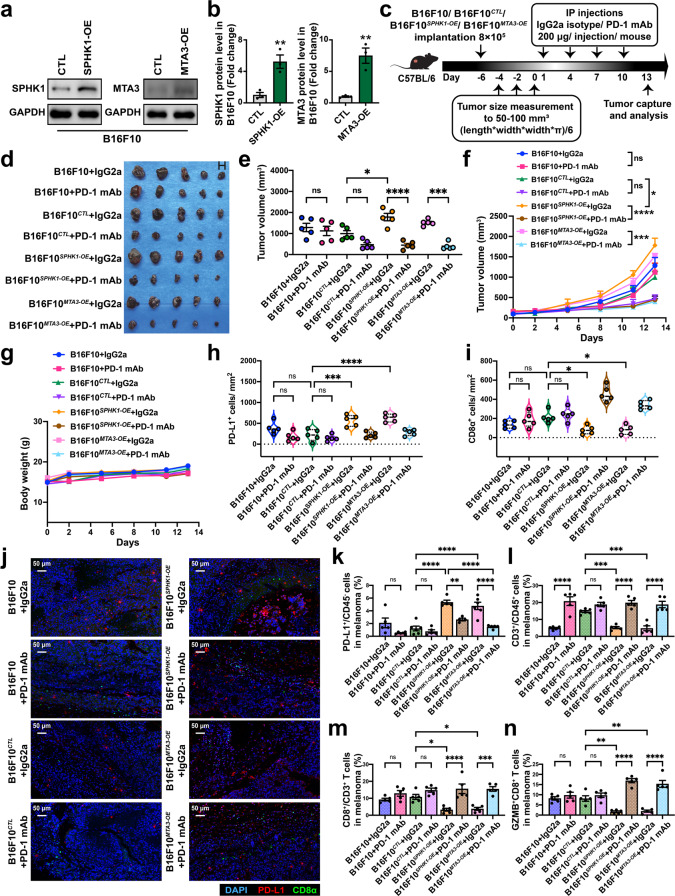
SPHK1-MTA3-PD-L1 axis-induced immune surveillance escape could be rescued by PD-1 mAb treatments in melanoma for B16F10 allograft immunocompetent mouse models. B16F10 melanoma cells were transfected with SPHK1, MTA3, or negative control (CTL) vector. SPHK1 and MTA3 expression was analyzed by western blot (**a**) and quantification (**b**). The plot (**b**) was generated from three independent experiments and showed as mean ± SEM. **c** Schematics of the treatment plan using a C57BL/6 female mouse model, established by subcutaneously injecting B16F10 overexpressing SPHK1, MTA3, or CTL. Mice were then either treated with PD-1 mAb (200 μg/ mouse), or IgG2a isotype control. **d** Image of B16F10 tumors, as captured on the thirteenth day. **e** Summary tumor volume data harvested on the thirteenth day. Plots of tumor volumes (**f**) and mice body weight (**g**) measured every two days. See also Supplementary Tables 7, 8. **h**, **i** Trichrome immunohistochemical staining of DAPI, CD8α, and PD-L1 expression of SPHK1-OE, MTA3-OE, or CTL B16F10 allografts. Violin plots indicating densities of PD-L1^+^ (**h**) and CD8α^+^ (**i**) cells/mm^2^ in IHC sections of B16F10 allografts. **j** Representative images, scale bars, 50 μm. Flow cytometric analyses were performed to measure PD-L1 on CD45^-^ subsets (**k**), CD3^+^ in CD45^+^ cells (**l**), CD8^+^ in CD3^+^ cells (**m**), and GZMB^+^CD8^+^ TILs (**n**) from B16F10 tumors. See also Supplementary Fig. 10. 5 mice per cohort. Data represent mean ± SEM. ns, non-significant, *p* > 0.05, *p < 0.05, **p < 0.01, ***p < 0.001, and ****p < 0.0001, as determined by unpaired two-sided Student’s *t*-test (**b**), one-way ANOVA and Tukey’s multiple comparisons test (**e**, **h**, **i**, **k**–**n**)

Next, we questioned whether SPHK1- or MTA3-mediated immune escape was reversed by ICB treatment in vivo. To this end, we first generated poorly immunogenic B16F10 mouse melanoma cells stably overexpressing SPHK1 or MTA3 (Fig. 5a, b). Then, an anti-mouse PD-1 mAb was utilized to treat immunocompetent mice inoculated with B16F10, B16F10^*SPHK1-OE*^, B16F10^*MTA3-OE*^, or B16F10^*CTL*^ cell lines (Fig. 5c). The analysis of mice bearing the B16F10 or B16F10^*CTL*^ melanoma revealed that PD-1 blockade therapies did not consistently inhibit tumor growth, which was consistent with published results [48–52]. SPHK1- or MTA3-OE cells showed significantly increased melanoma growth compared with B16F10^*CTL*^ cells. Intriguingly, we observed that SPHK1- or MTA3-induced tumor proliferation was substantially suppressed by anti-PD-1 mAb treatment, as demonstrated by a reduction in the tumor growth rate (Fig. 5d–g and Supplementary Tables 7, 8). Multiplex IHC staining showed that overexpression of SPHK1 or MTA3 significantly boosted PD-L1^+^ cells but decreased the density of CD8^+^ T cells in the tumor region, indicating that SPHK1-MTA3-PD-L1 axis-mediated immune escape contributes to melanoma progression (Fig. 5h–j and Supplementary Fig. 10k). Flow cytometric analyses showed that overexpression of SPHK1 or MTA3 markedly enhanced the expression of PD-L1 on CD45^-^ subsets (Fig. 5k and Supplementary Fig. 10a). Moreover, the percentage of infiltrating CD3^+^ T lymphocytes in the CD45^+^ population (Fig. 5l), as well as the percentage of CD8^+^ T cells in the CD3^+^ T-cell population (Fig. 5m), was significantly repressed in tumor tissues. Furthermore, we found that GZMB^+^CD8^+^ T cells were significantly decreased in the SPHK1- and MTA3-OE groups compared with the control group, which was consistent with our previous findings (Fig. 5n). Importantly, the results showed that compared with IgG2a treatments, PD-1 blockade therapies significantly reversed SPHK1- or MTA3-associated immune dysfunctions and enhanced the immune response against tumor cells, as reflected by the frequencies of CD8^+^ and GZMB^+^CD8^+^ T lymphocytes in melanoma. We assessed the expression of PD-1 on CD45^+^ cell subsets and found that PD-1 blockade led to a slight reduction in the frequencies of PD-1^+^CD45^+^ cells (Supplementary Fig. 10b, e). In contrast, before treatment with PD-1 mAb, the frequency of PD-1^+^CD8^+^ T cells was increased 1- to 2-fold higher in the B16F10^*SPHK1-OE*^ and B16F10^*MTA3-OE*^ groups versus that in the B16F10^*CTL*^ group (Supplementary Fig. 10f). As PD-1 on CD8^+^ T-cell subsets is a novel identified marker in predicting the response to anti-PD-1 immunotherapies, these data suggest that SPHK1 and MTA3 may serve as potential biomarkers for the prognosis of melanoma and the efficacy of PD-1 mAb blockade therapies [53–56]. We then detected the abundance of immunosuppressive cells in the B16F10^*SPHK1-OE*^ and B16F10^*MTA3-OE*^ groups. The results showed that the percentage of MDSC subsets in the group of PD-1 blockade treated-B16F10^*SPHK1-OE*^ or B16F10^*MTA3-OE*^ was significantly lower than that in the B16F10^*CTL*^ group (Supplementary Fig. 10g, h). Notably, the SPHK1-MTA3 axis enhanced the tumor infiltration of CD4^+^CD25^+^FoxP3^+^ Tregs and F4/80^+^CD11b^+^CD206^+^MHC class II^-^ M2-like tumor-associated macrophages, and this effect could be reversed by PD-1 mAb treatment (Supplementary Fig. 10b–d, i, j). Taken together, these data reveal a novel strategy through which SPHK1-MTA3-PD-L1 axis-mediated immune tolerance and tumor proliferation can be rescued by anti-PD-1 immunotherapies.

### The SPHK1 and MTA3 expression levels are correlated with the therapeutic efficacy and prognosis of anti-PD-1 mAb treatment in melanoma patients

**Fig. 6:**
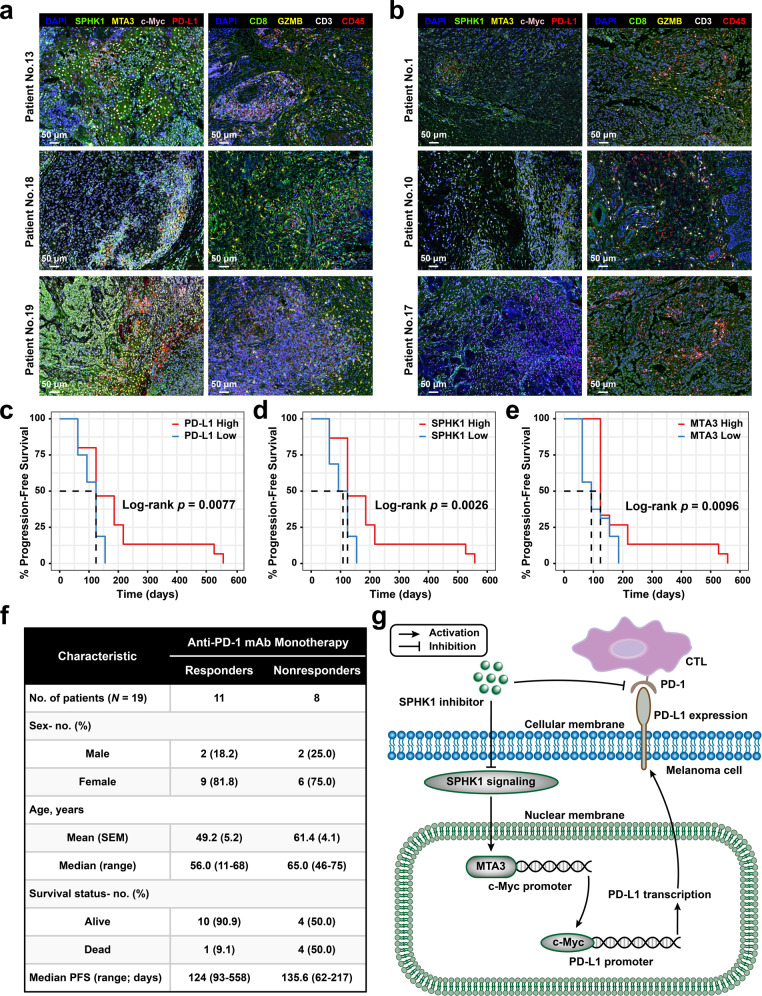
The expression of SPHK1 and MTA3 is related to the prognosis of anti-PD-1 therapy in melanoma patients. **a**, **b** Six melanoma patients including responders (No.13, No.18, No.19) and nonresponders (No.1, No.10, No.17) were visualized by multiplex IHC staining of DAPI, SPHK1, MTA3, c-Myc, PD-L1, CD8, GZMB, CD3, and CD45. Scale bars, 50 μm. **c**–**e** Kaplan–Meier survival curves of melanoma patients’ progression-free survival. Patients were stratified into two groups by median gene expression. Significance was determined by the Log-rank test. **f** Clinicopathologic characteristics of anti-PD-1 mAb monotherapy cohorts. SEM, standard error of mean, PFS, progression-free survival. All patients were treated with PD-1 monoclonal antibody after surgical resection of primary lesions, and those with recurrence or metastasis ≤6 months were considered as nonresponders (NR), while those without recurrence or metastasis longer than 6 months were considered as responders (R). See also Supplementary Table 9. **g** Schematic model showing the mechanism and role of SPHK1 in melanoma cells

To further evaluate the impact of MTA3 on the therapeutic effect of anti-PD-1 immunotherapy, we compared melanoma responders and nonresponders undergoing PD-1 blockade therapy from the immune checkpoint blockade datasets of the Gene Expression Omnibus (GEO) database and calculated the expression of MTA3 within each group. The results showed that MTA3 was linked to a positive response to anti-PD-1 immunotherapy, suggesting the potential significance of MTA3 in the prognosis of immunotherapy (Supplementary Fig. 11a). To assess the prognostic features of MTA3 in melanoma patients, we extracted time-to-event data and conducted survival analyses. We noted no significant differences in the risk of death between patients with high or low MTA3 expression who were not treated with immunotherapies (hazard ratio [HR] 1.20, 95% confidence interval [CI] 0.87–1.64; Supplementary Fig. 11b). Conversely, patients with high MTA3 expression who received immunotherapies in the group with high MTA3 expression had a significantly decreased risk of death (HR 0.40, 0.19–0.85; Supplementary Fig. 11c). Furthermore, we delineated the expression of SPHK1, MTA3, c-Myc, and PD-L1 and tumor-infiltrating immune cell profiles in melanoma biopsies from a total of nineteen patients regularly receiving PD-1 blockade therapies (toripalimab; Fig. 6a, b and Supplementary Fig. 11d, e). Among the recruited patients, eight patients with disease progression were identified as nonresponders, and the other eleven patients with a positive response to PD-1 blockade therapies were classified as responders (Fig. 6f and Supplementary Table 9). Correlation analyses suggested a significant difference in PD-L1 membrane expression between the melanoma population with high or low cytoplasmic expression of SPHK1 (*p* = 9.85 × 10^−4^), or with high or low nuclear expression of MTA3 (*p* = 5.48 × 10^−3^; Supplementary Table 10). Furthermore, we observed that melanoma patients treated with PD-1 blockade therapies with high SPHK1 (HR 0.30, 0.13–0.72), high MTA3 (HR 0.44, 0.20–0.95), high c-Myc (HR 0.16, 0.052–0.48) or high PD-L1 (HR 0.33, 0.14–0.80) expression levels in tumors exhibited prolonged progression-free survival (PFS; Fig. 6c-e and Supplementary Fig. 11f). Together, these preclinical results demonstrated that high expression of SPHK1 has the potential to reverse resistance to anti-PD-1 mAb therapy induced by upregulated PD-L1 expression in tumors and that the SPHK1-MTA3 axis could serve as a potential biomarker for the prognosis of melanoma patients receiving anti-PD-1 immunotherapies.

In summary, we elucidated an emerging functionality of SPHK1, identified SPHK1-mediated immunosuppression established by transcriptional regulation of PD-L1 in malignant melanoma and demonstrated a novel mechanism of PD-L1 expression. Importantly, the SPHK1-MTA3 axis holds the potential to predict melanoma patient outcomes in response to anti-PD-1 mAb therapies.

## Discussion

Herein, we elucidated the molecular mechanism underlying how SPHK1 transcriptionally regulates tumor PD-L1 expression. In addition, MTA3 was identified as a vital target of SPHK1 and was found to participate in the regulation of PD-L1 expression. Our discoveries also illustrated that SPHK1-MTA3 maintains the immunosuppressive status in the TME and promotes tumor immune escape by positively regulating PD-L1 on melanoma cells, decreasing the proportion of TILs, and inhibiting tumor-specific CTL activation in an independent manner. Importantly, melanoma cells with high SPHK1 and MTA3 expression were found to be highly sensitive to anti-PD-1 mAb-mediated tumor cytotoxic effects in vivo. SPHK1 and MTA3 hold the potential to function as novel biomarkers to predict melanoma patient outcomes upon anti-PD-1 mAb blockade therapy.

These findings are noteworthy for the following reasons. First, we showed a new and undiscovered association between SPHK1 and PD-L1, which are crucial molecules in tumor occurrence and development, particularly in malignant melanoma with high immunogenicity. Furthermore, the role of SPHK1 in cancers has gained considerable attention, and SubbaRao and colleagues have previously showed that targeting SPHK1 decreases pAkt expression and arrests cells in the G0/G1 phase of the melanoma cell cycle, which results in the reduction of tumor cell proliferation and induction of apoptosis [57]. In addition, the data from Albinet and colleagues have introduced the concept that SPHK1 could initiate fibroblast differentiation in melanoma and lead to tumor metastasis [58]. The data reported by Caroline Imbert and colleagues have shown that combination strategies involving SPHK1 inhibition enhance ICB therapy by inhibiting immunosuppressive cells in tumors [59]. Consistent with these studies, we have revealed the role of SPHK1 in the oncogenesis and process of melanoma, and further filled a knowledge gap of paramount importance regarding SPHK1-mediated immune escape in melanoma. In addition, MTA3 was first found to be a potential target of SPHK1 and to participate in the transcriptional regulation of PD-L1 in malignant melanoma.

MTA3 has been identified as a constituent of the nucleosome remodeling and deacetylase (NuRD) complex and performs a structural function in forming the histone deacetylase core subcomplex (HDCC) of NuRD [60–62]. On the one hand, the biological characteristics of MTA3 in breast cancer have been recognized. Accumulating studies have demonstrated that estrogen receptor-α (ER-α) can bind to the promoter of MTA3 to directly regulate transcription [22, 63–65]. In this study, we observed the expression of MTA3 in melanoma cells and proved that MTA3 boosted melanoma progression in vivo. Importantly, the role of MTA3 in stabilizing immunosuppression in the TME provides new insights for future investigation of and clinical therapeutics in melanoma.

PD-L1 regulation has emerged as an important topic due to the multiple functions of PD-L1 in the TME and cancer outcomes. To our knowledge, melanoma is characterized by a relatively high TMB and PD-L1 expression. Tumor PD-L1 expression is mediated not only by endogenous oncogenes but also by the more important mechanism of PD-L1 induction by IFN-γ secreted from immune cells [66–68]. CD8^+^T cells upregulate PD-1 expression and secrete IFN-γ after recognition of tumor antigens presented by antigen-presenting cells [69]. Partially dysfunctional PD-1^+^CD8^+^ T-cell subsets have been proven to be capable of producing IFN-γ and further upregulating the expression of PD-L1, which results in creation of a negative feedback loop that eliminates immune surveillance and sustains immunosuppression [53–55]. In this study, we aimed to explore the regulation of IFN-γ-induced PD-L1 expression by SPHK1 to mimic the immune microenvironment in the vicinity of tumor cells. Moreover, targeting SPHK1 has been demonstrated to reverse adaptive immune resistance established by T-cell-induced persistent PD-L1 expression and further activate the T-cell-mediated antitumor response. In the present study, we found that SPHK1-MTA3 axis-induced PD-L1 expression in melanoma could improve anti-PD-1 therapy in vivo, which indicated that the SPHK1-MTA3 axis might be a potential biomarker for the prediction of the prognosis of advanced melanoma patients. Previous studies have revealed the use of PD-L1 on tumor cells as a potential biomarker to identify patients with non-small-cell lung cancer who are most likely to benefit from PD-1/PD-L1 blockade therapy (i.e., pembrolizumab, atezolizumab, and nivolumab) [70–73]. The FDA has approved PD-L1 expression determined by immunohistochemistry as a companion diagnostic for anti-PD-1/PD-L1 antibody treatment in advanced non-small-cell lung cancer. The indications for the predictive capacity of PD-L1 expression in other cancer types have been under intensive investigation. Given the complexity of the tumor microenvironment, other components of tumor cells might be valuable as predictive biomarkers together with PD-L1 expression for the accurate selection of optimal treatments for cancer patients.

Our observations are also significant due to the potential value of the SPHK1-MTA3 axis in predicting the prognosis of melanoma patients receiving PD-1 blockade treatments. We speculated that the SPHK1-MTA3 axis-mediated high expression of PD-L1 in melanoma promotes the adaptive immune resistance and that this dysfunction can be rescued by PD-1 blockade. Previous studies have highlighted the importance of PD-1^+^CD8^+^ T cells in the TME and revealed that high percentages of PD-1^+^CD8^+^ T-cell subsets are strongly correlated with the efficacy of ICIs. In addition, PD-1^+^CD8^+^ T-cell populations have been confirmed to be higher in patients who respond to anti-PD-1 therapies than in nonresponders [53, 54, 74–76]. Based on these results, we found that the existing immunosuppression created by the SPHK1-MTA3 axis could be reversed by anti-PD-1 mAb treatments, as demonstrated by the accumulation of tumor-infiltrating PD-1^+^CD8^+^ T cells.

We have elucidated an emerging functionality of SPHK1, identified SPHK1-mediated immunosuppression established by transcriptional regulation of PD-L1 in malignant melanoma and demonstrated a novel mechanism of PD-L1 expression. Importantly, the SPHK1-MTA3 axis holds the potential to predict melanoma patient outcomes in response to anti-PD-1 mAb therapies.

## Supplementary information

Supplementary Information

## Data Availability

All data generated or analyzed during this study are included in this published article.
