# Supplementary material for: Sphingosine kinase 1 promotes tumor immune evasion by regulating the MTA3-PD-L1 axis
Source: Cell Mol Immunol. 2022 Sep 1;19(10):1153–67. doi: 10.1038/s41423-022-00911-z (PMC9508236; doi:10.1038/s41423-022-00911-z)
Supplement: Supplementary file 1 — Supplementary Information [file 41423_2022_911_MOESM1_ESM.pdf]

Supplementary Information

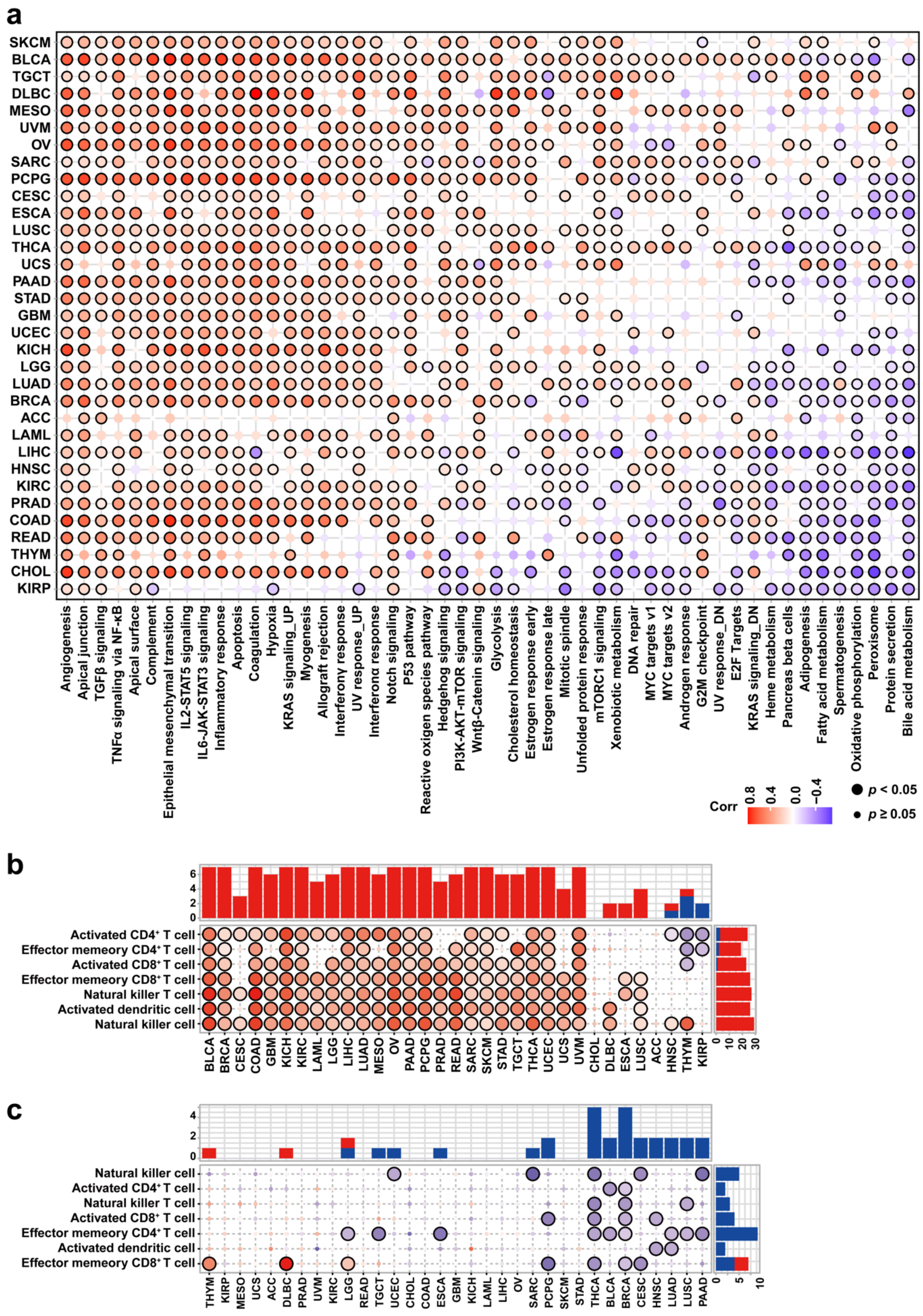

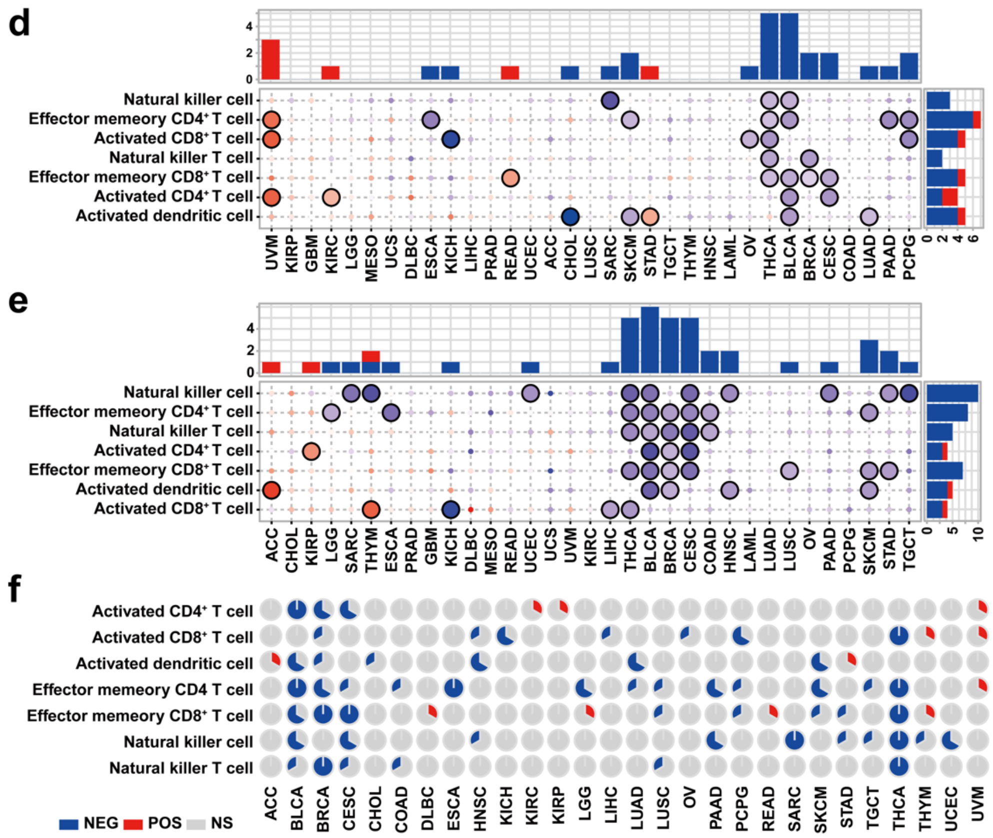

Supplementary Fig. 1, related to Fig. 1.

a, Spearman's correlation between SPHK1 expression and MSigDB hallmark pathways across 33 cancer types. **b,** Spearman's correlation between SPHK1 expression and anti-tumor immune cells. **c-f,** Spearman's correlation between SPHK1 expression and ratio of anti-tumor and suppressive immune cells (**c-e**), and the fraction of significantly correlation (**f**). Color of dots represent the correlation. Blackmark represents the significance (Spearman's correlation test, $p < 0.05$). Color of bar plots and pie charts represent the count and fraction of significant correlation. Blue represents a significantly negative correlation (NEG), red represents a significantly positive correlation (POS), and grey represents non-significant correlation (NS).

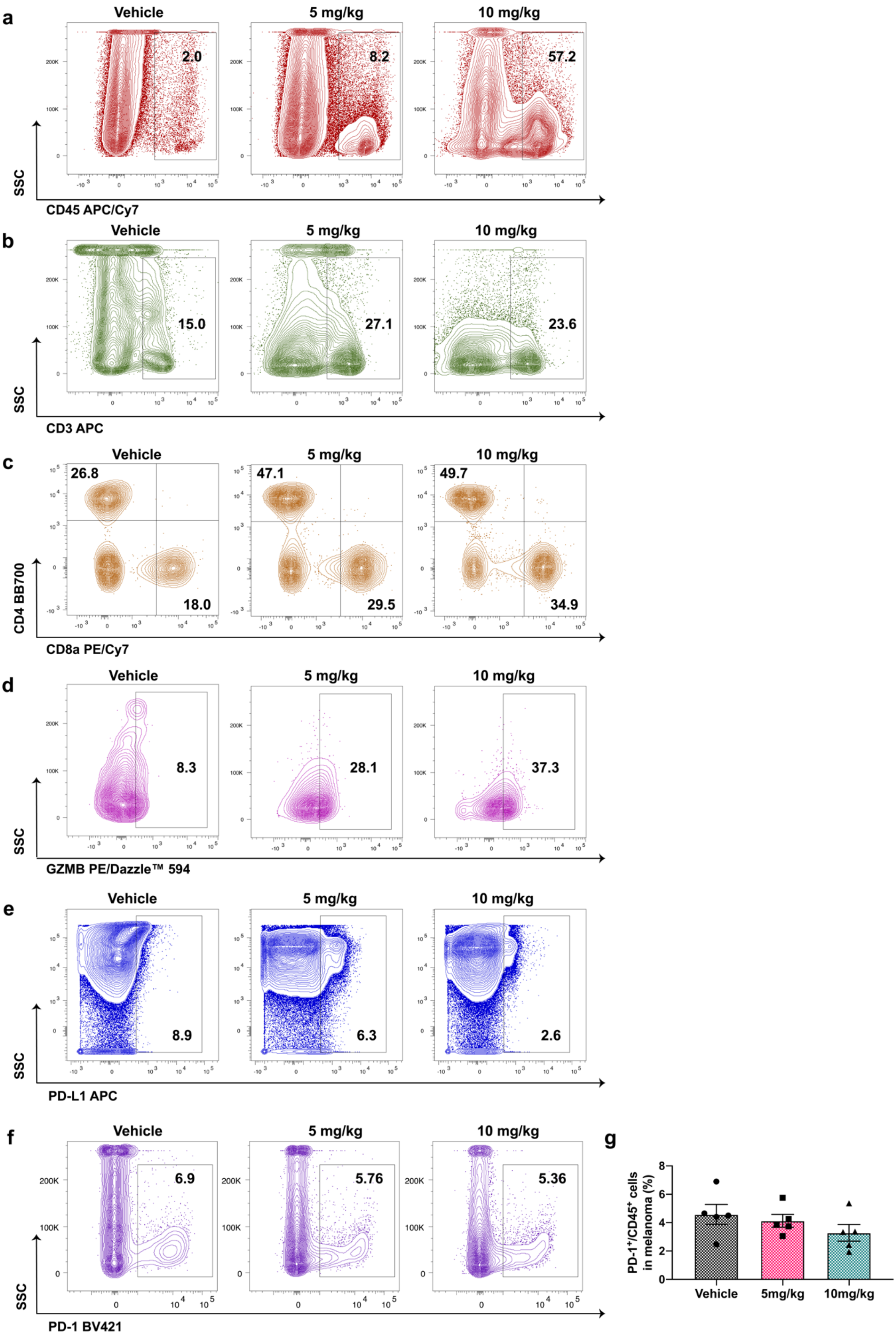
a
Vehicle
5 mg/kg
10 mg/kg
2.0
8.2
57.2
SSC
CD45 APC/Cy7
b
Vehicle
5 mg/kg
10 mg/kg
15.0
27.1
23.6
SSC
CD3 APC
c
Vehicle
5 mg/kg
10 mg/kg
26.8
18.0
47.1
29.5
49.7
34.9
CD4 BB700
CD8a PE/Cy7
d
Vehicle
5 mg/kg
10 mg/kg
8.3
28.1
37.3
SSC
GZMB PE/Dazzle™ 594
e
Vehicle
5 mg/kg
10 mg/kg
8.9
6.3
2.6
SSC
PD-L1 APC
f
Vehicle
5 mg/kg
10 mg/kg
6.9
5.76
5.36
SSC
PD-1 BV421
g
PD-1+/CD45+ cells in melanoma (%)
Vehicle
5mg/kg
10mg/kg

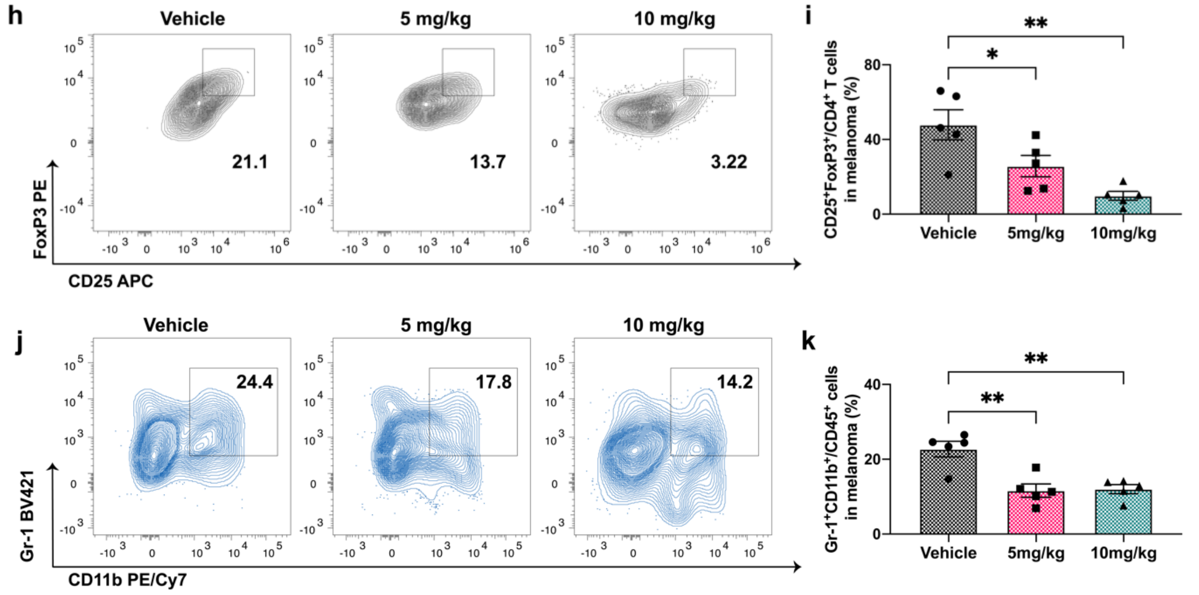

Supplementary Fig. 2, related to Fig. 2.

a-e, Representative profiles of fluorescence-activated cell sorting of CD45^{+} cells (**a**), CD3^{+} in CD45^{+} cells (**b**), CD4^{+} or CD8^{+} in CD3^{+} cells (**c**), GZMB^{+}CD8^{+} TILs (**d**), and PD-L1 on CD45^{-} cells (**e**) from B16F10. **f-g,** Fluorescence-activated cell sorting (FACS) of PD-1^{+}CD45^{+} cells from B16F10 plots (**f**) and quantification (**g**). **h-i,** FACS analysis of CD25^{+}FoxP3^{+} in CD4^{+} T cells plots (**h**) and quantification (**i**). **j-k,** FACS analysis of Gr-1^{+}CD11b^{+} in CD45^{+} cells plots (**j**) and quantification (**k**). Data represent mean ± SEM. ns, non-significant, $p > 0.05$, *$p < 0.05$, **$p < 0.01$, and ***$p < 0.001$, as determined by one-way ANOVA and Tukey's multiple comparisons test (**g, i, k**).

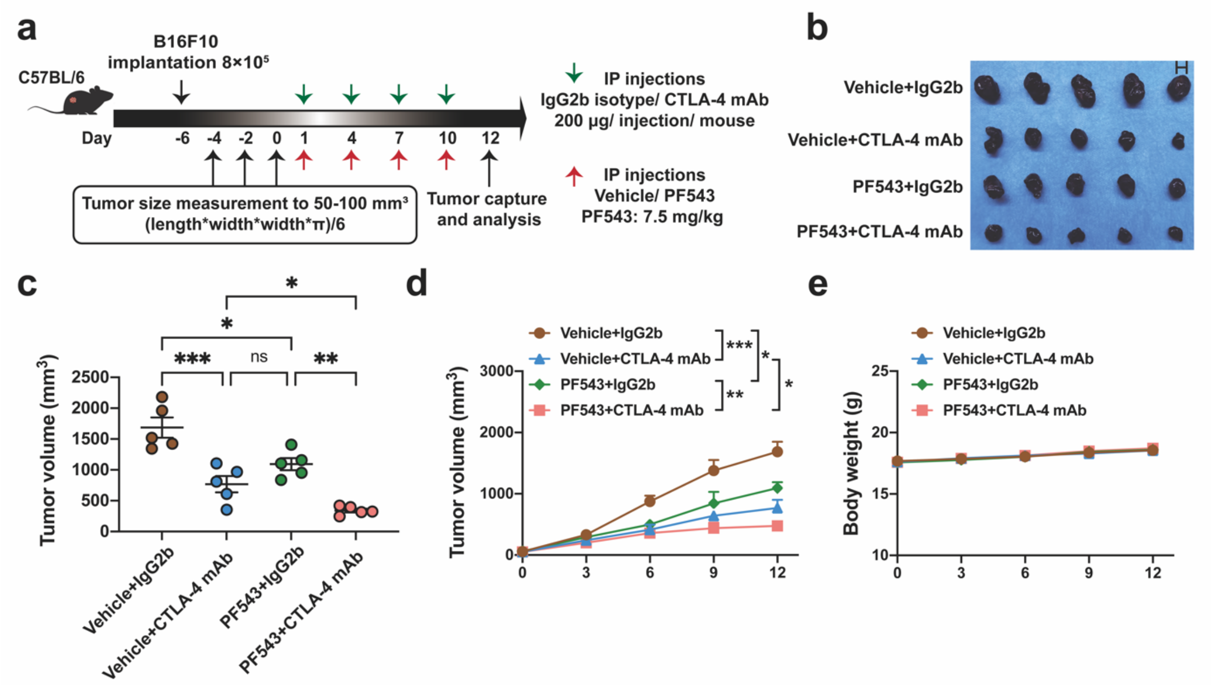

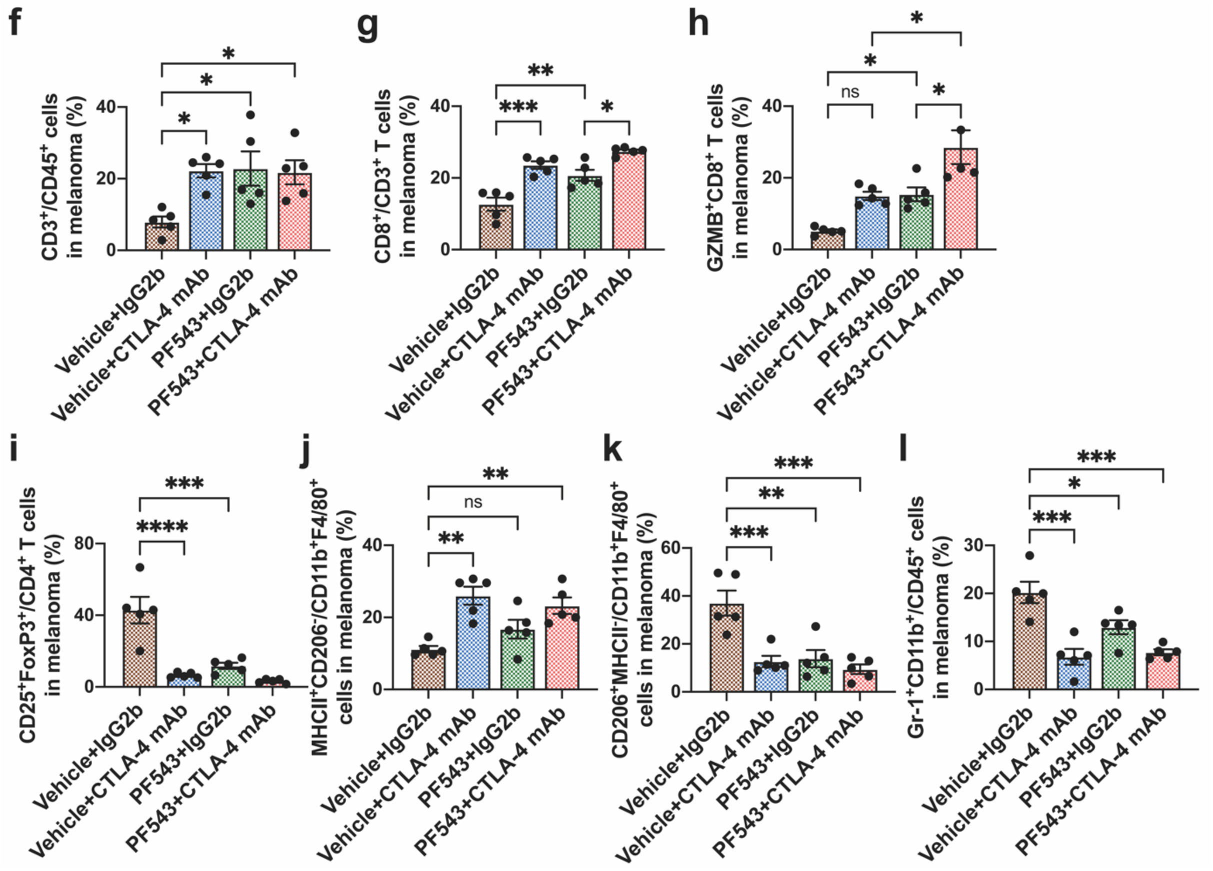
f
CD3⁺/CD45⁺ cells in melanoma (%)
g
CD8⁺/CD3⁺ T cells in melanoma (%)
h
GZMB⁺CD8⁺ T cells in melanoma (%)
i
CD25⁺FoxP3⁺/CD4⁺ T cells in melanoma (%)
j
MHCII⁺CD206⁻/CD11b⁺F4/80⁺ cells in melanoma (%)
k
CD206⁺MHCII⁻/CD11b⁺F4/80⁺ cells in melanoma (%)
l
Gr-1⁺CD11b⁺/CD45⁺ cells in melanoma (%)
Vehicle+IgG2b
Vehicle+CTLA-4 mAb
PF543+IgG2b
PF543+CTLA-4 mAb
ns

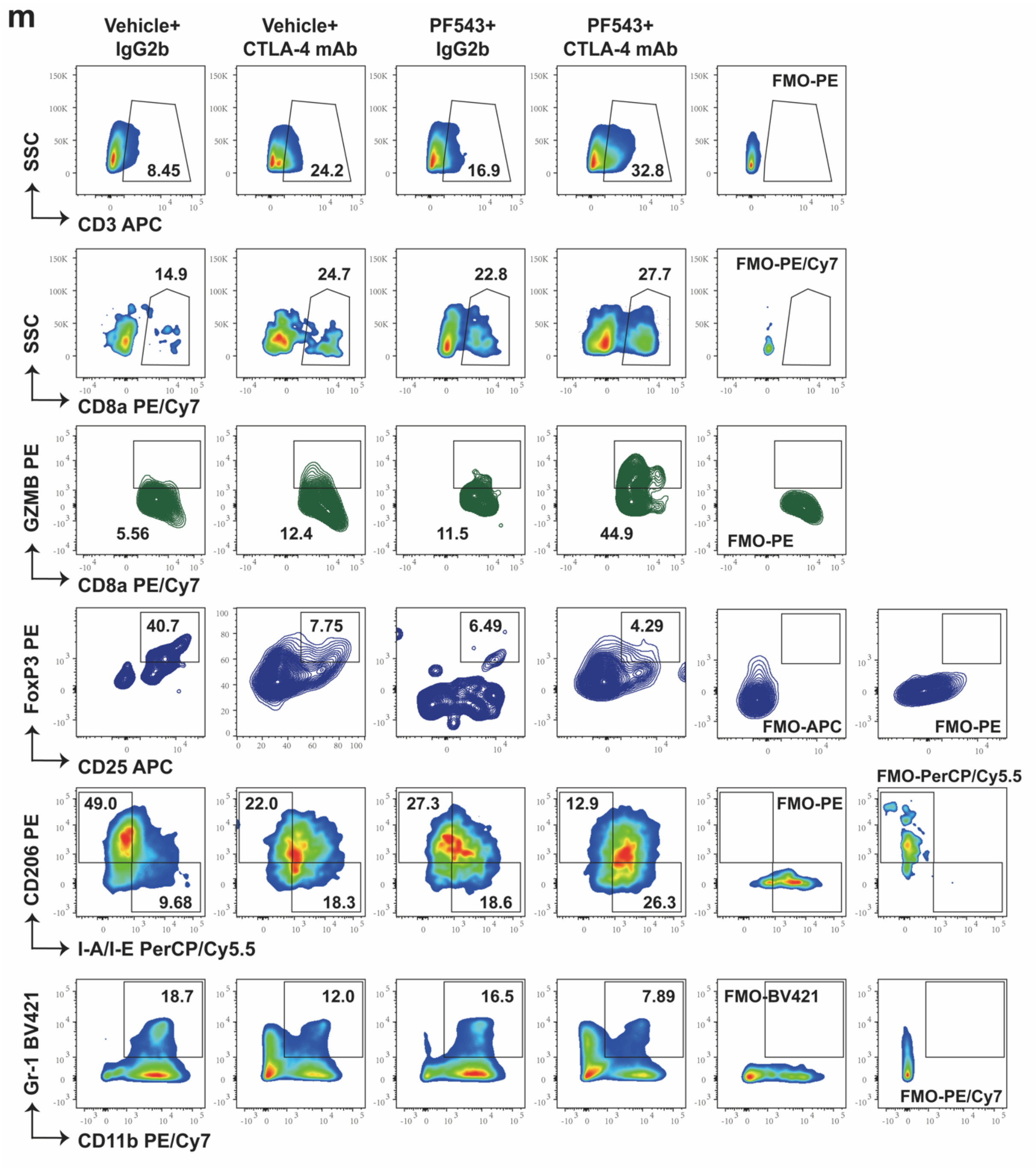

Supplementary Fig. 3 The combination of PF543 and CTLA-4 mAb effectively suppresses melanoma tumor growth *in vivo*.

a, Schematic diagram illustrating the treatment protocol of CTLA-4 mAb or/ and PF543 in B16F10 cells constructed mice model. At the endpoint, tumor cells were isolated for analysis. **b,** Image of B16F10 tumors, as captured on the twelfth day. **c,** Summary tumor volume data harvested on the twelfth day. See also Supplementary Tables 3-4. **d-e,** B16F10 tumor volume (**d**) and body weight (**e**) were measured at the indicated time points. **f-m,** FACS quantification of $CD3^+$ in $CD45^+$ cells (**f**), $CD8^+$ (**g**), $GZMB^+CD8^+$ (**h**) in $CD3^+$ cells, $CD25^+FoxP3^+$ in $CD4^+$ T cells (**i**), MHC class II^+CD206^- in $CD11b^+F4/80^+$ cells (**j**), $CD206^+$MHC class II^- in $CD11b^+F4/80^+$ cells (**k**), $Gr\text{-}1^+CD11b^+$ in $CD45^+$ cells (**l**) and plots (**m**). 5 mice per cohort. Data represent mean ± SEM. ns, non-significant, $p > 0.05$, $*p < 0.05$, $**p < 0.01$, and $***p < 0.001$, as determined by one-way ANOVA and Tukey's multiple comparisons test (**c, f-l**).

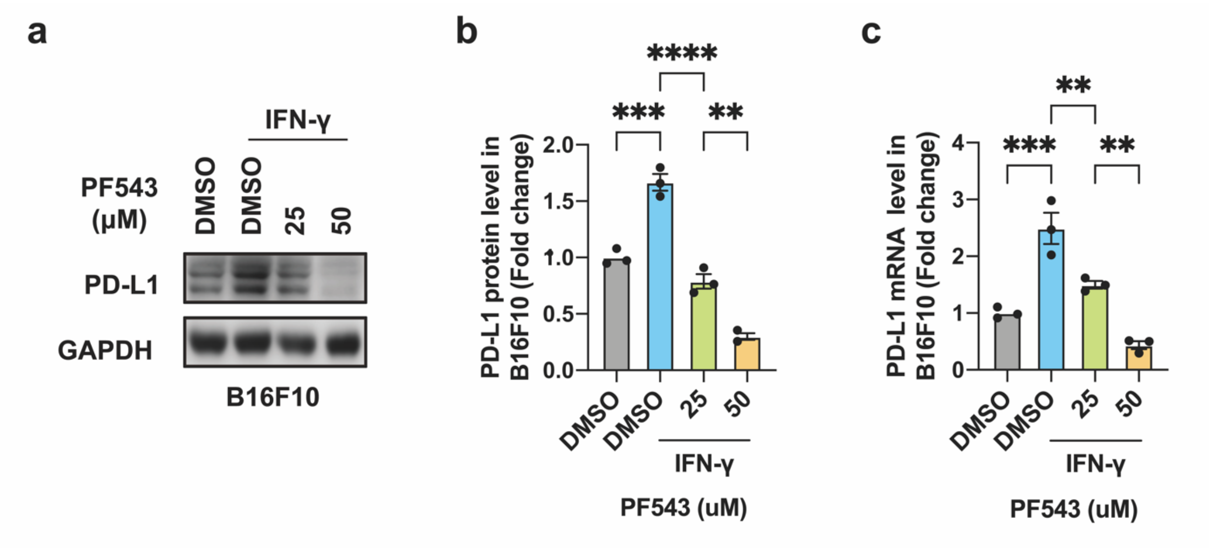

Supplementary Fig. 4 PF543 treatment associates with decrease of tumor PD-L1 in B16F10 *in vitro*.

a, Representative Western blot analysis of B16F10 cell line treated with increasing concentrations of PF543 (25 μM, 50 μM) for 24 hours under IFN-γ exposure. **b,** Bar graph presenting the quantitative analysis of PD-L1 protein expression data from (**a**). **c,** Bar diagram presentation of PD-L1 mRNA levels as determined by qRT-PCR of increasing concentrations of PF543 treatment for 24 hours under IFN-γ exposure (n = 3). The plot (**b**) was generated from three independent experiments and showed as mean ± SEM. ns, non-significant, $p > 0.05$, *$p < 0.05$, **$p < 0.01$, ***$p < 0.001$, and ****$p < 0.0001$, as determined by one-way ANOVA and Tukey's multiple comparisons test (**b, c**).

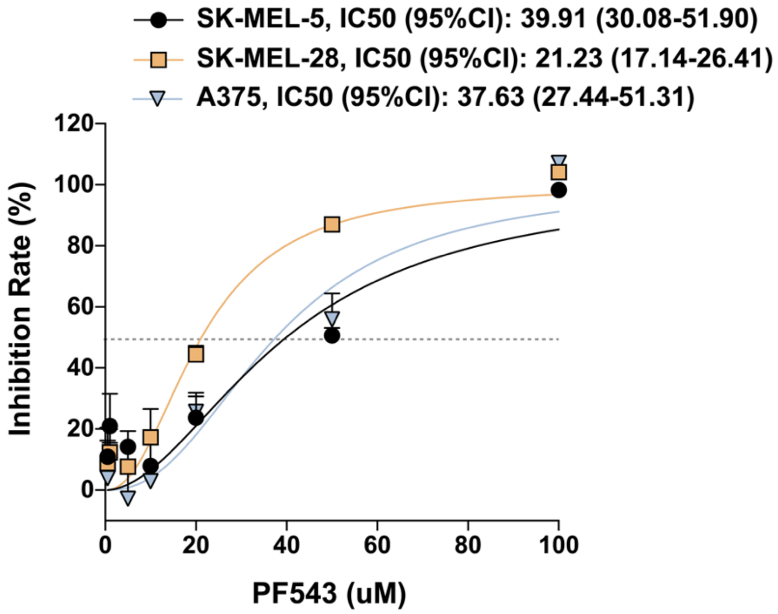

Supplementary Fig. 5 Dose-response to PF543 in melanoma cell lines.

SK-MEL-5, SK-MEL-28, and A375 melanoma cells were treated with DMSO or different concentrations of PF543 for 24 hours. Data represent mean ± SEM. IC50, half maximal

inhibitory concentration.

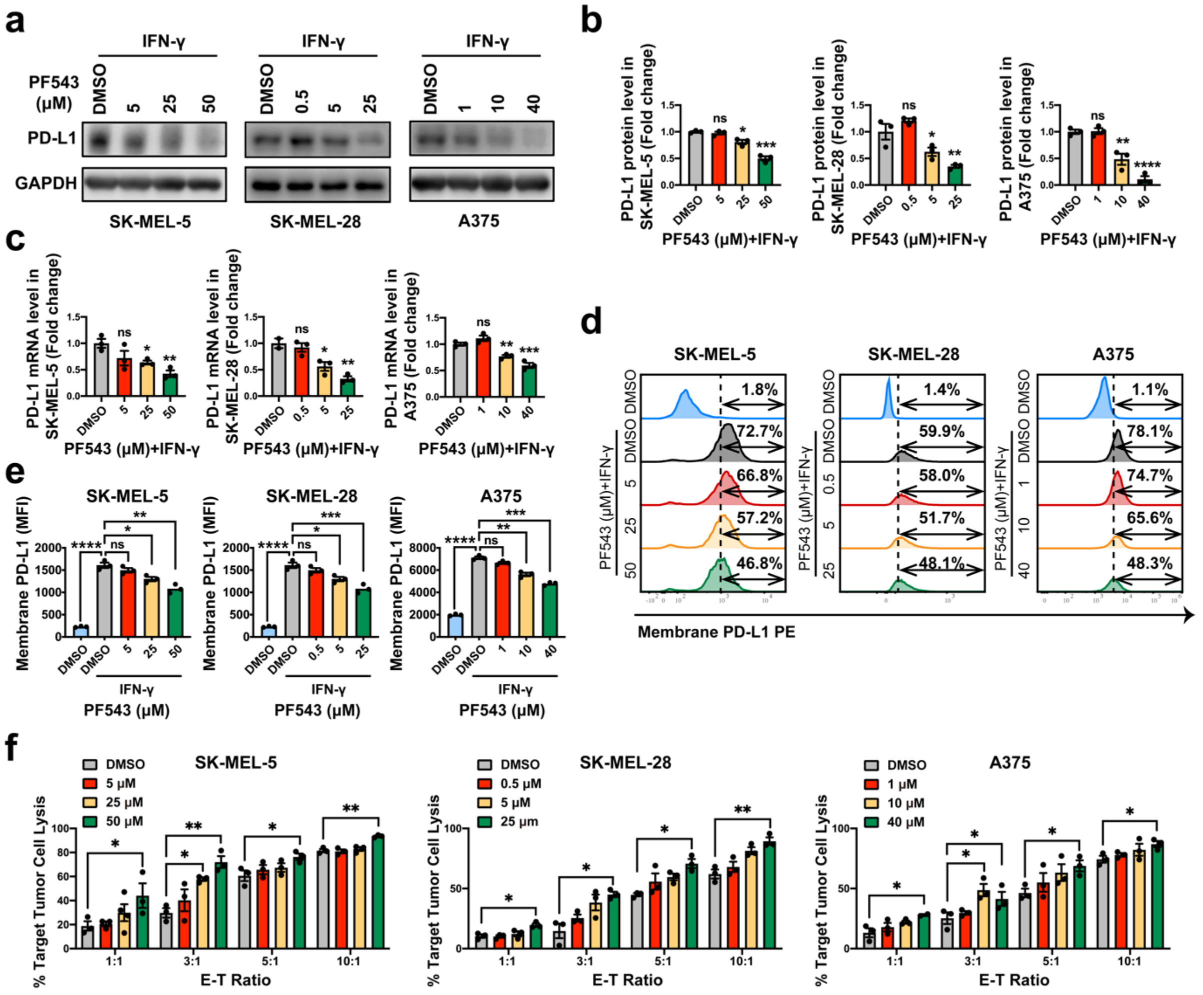

Supplementary Fig. 6 SPHK1 inhibitor PF543 leads to the suppression of tumor growth and correlates with the expression of tumor PD-L1 *in vitro*.
a-c, PD-L1 expression in SK-MEL-5, SK-MEL-28, and A375 cells treated with PF543 (concentrations based on dose-response analysis, see also Supplementary Fig. 5) and IFN-γ (200 ng/mL) for 24 hours was analyzed by western blot (**a**) and quantification (**b**), or by RT-PCR (**c**; n = 3). The plot (**b**) was generated from three independent experiments and showed as mean ± SEM. **d,** Representative profiles of flow cytometric analysis of PD-L1^{+} membrane expression. **e,** Quantitative analysis of membrane PD-L1 of melanoma cell lines by mean fluorescence intensity (MFI). **f,** T cell-mediated tumor cell killing assay, SK-MEL-5, SK-MEL-28, and A375 cells treated with PF543 or DMSO co-cultured with activated T cells for 24 hours. The viability of tumor cells was analyzed with CCK8 assays (n = 4). Data are presented as mean ± SEM. ns, non-significant, $p > 0.05$, *$p < 0.05$, **$p < 0.01$, ***$p < 0.001$, and ****$p < 0.0001$, as determined by Dunnett's multiple comparisons test (**b, c, e**) and two-way ANOVA (**f**).

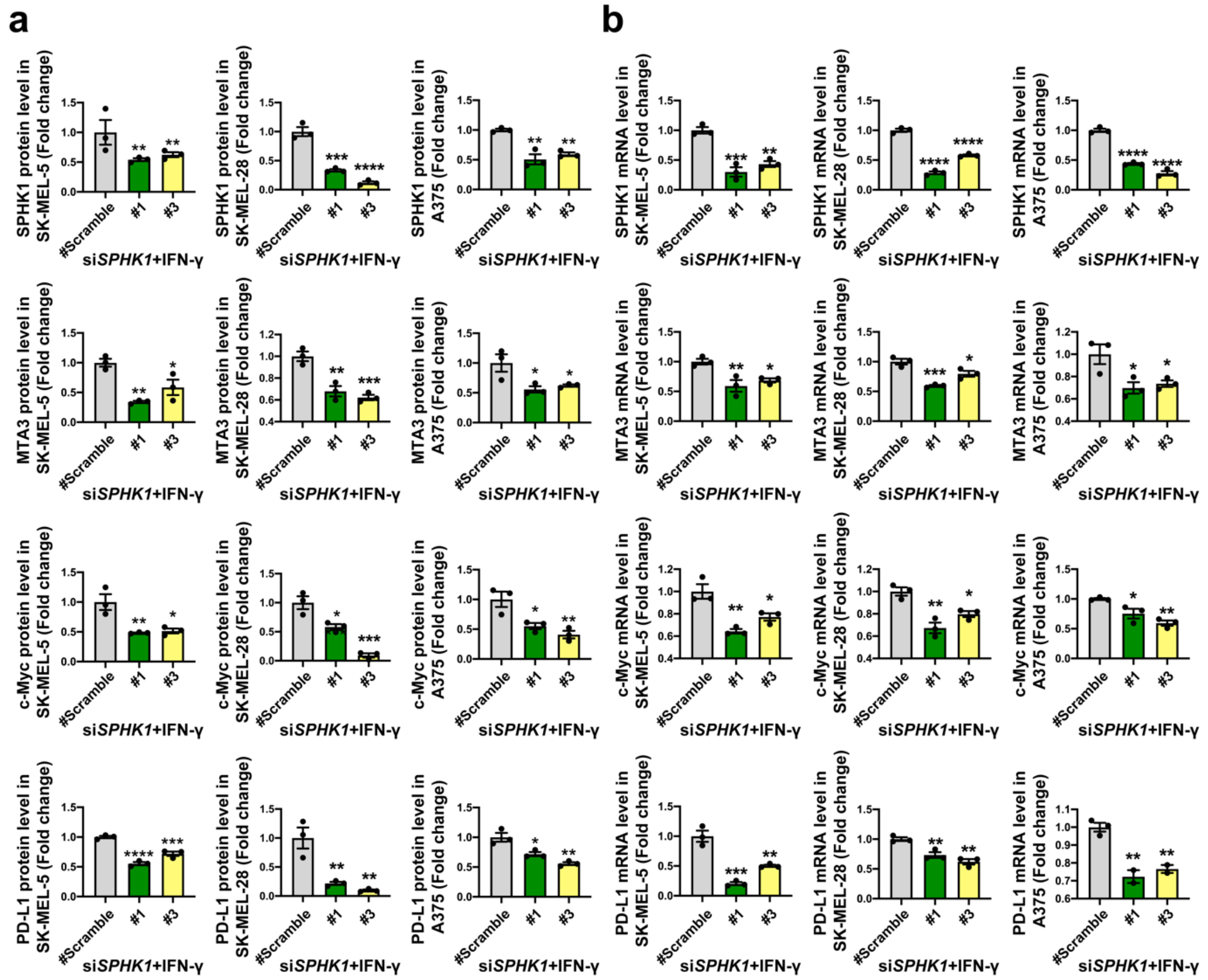

Supplementary Fig. 7, related to Fig. 4.

a-b, SK-MEL-5, SK-MEL-28, and A375 melanoma cells were transfected with si*SPHK1* or scrambled negative control si*RNA*. SPHK1, MTA3, c-Myc, or PD-L1 expression was analyzed by western blot (**a**) and RT-PCR (**b**; n = 3). The plot (**a**) was generated from three independent experiments and showed as mean ± SEM. ns, non-significant, $p > 0.05$, *$p < 0.05$, **$p < 0.01$, ***$p < 0.001$, and ****$p < 0.0001$, as determined by Dunnett's multiple comparisons test.

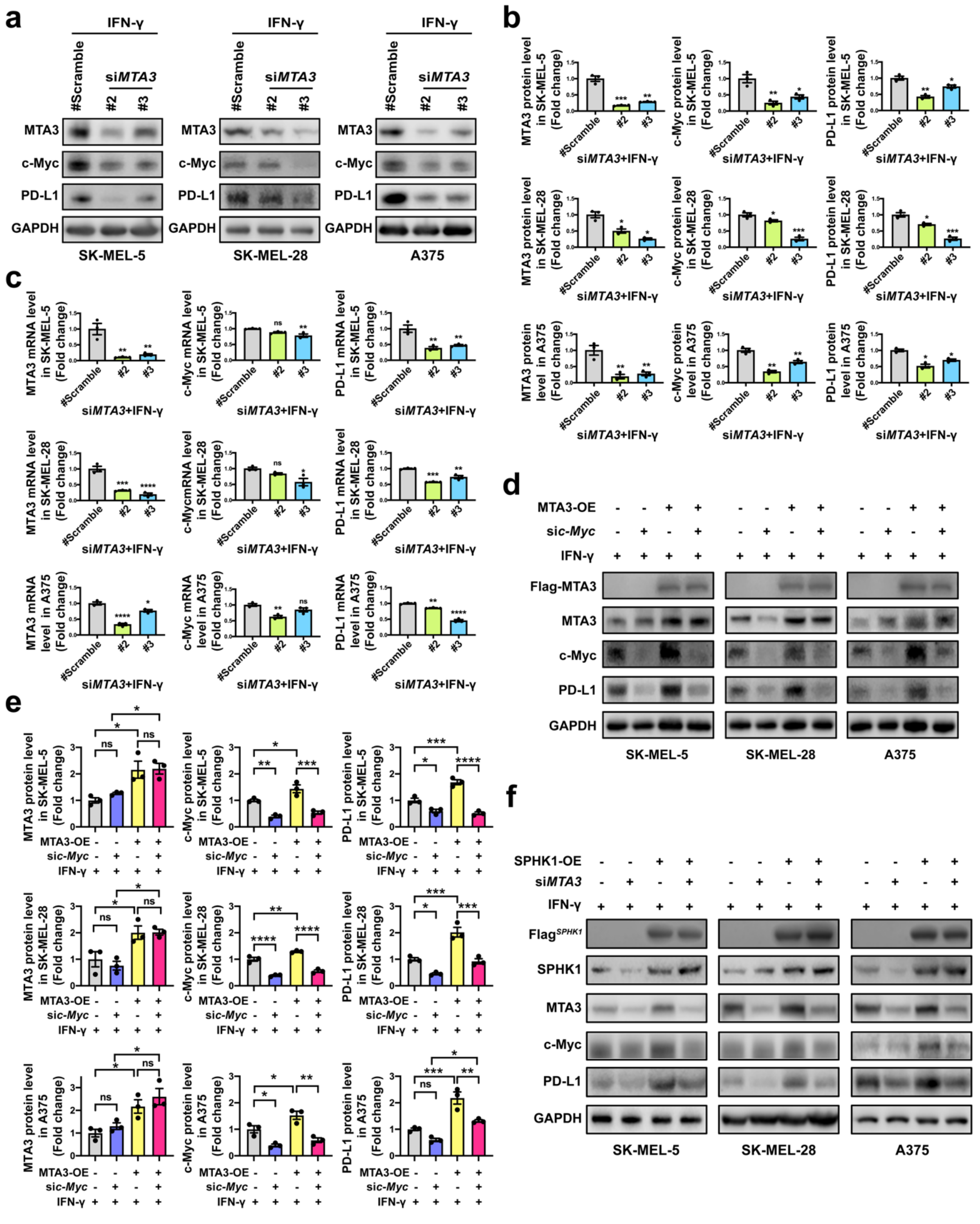

Supplementary Fig. 8 SPHK1-MTA3 axis regulated PD-L1 expression levels *in vitro*.

a-c, Melanoma cells were transfected with si*MTA3* or scrambled negative control si*RNA*. MTA3, c-Myc, or PD-L1 expression were visualized and analyzed by western blot (**a**) and quantification (**b**), or by RT-PCR (**c**; n = 3). The plot (**b**) was generated from three independent experiments and showed as mean ± SEM. **d-e,** SK-MEL-5, SK-MEL-28, and A375 cells overexpressing MTA3 were transfected with sic-*Myc* or scrambled negative control si*RNA*. MTA3, c-Myc, or PD-L1 expression was visualized by western blot (**d**) and quantification (**e**). The plot (**e**) was generated from three independent experiments and showed as mean ± SEM. **f,** Melanoma cells overexpressing SPHK1 were transfected with si*MTA3* or scrambled negative

control si*RNA*, SPHK1, MTA3, c-Myc, or PD-L1 expression was analyzed by western blot. Data represent mean ± SEM. ns, non-significant, $p > 0.05$, *$p < 0.05$, **$p < 0.01$, ***$p < 0.001$, and ****$p < 0.001$, as determined by Dunnett's multiple comparisons test (**b, c**) and Tukey's multiple comparisons test (**e**).

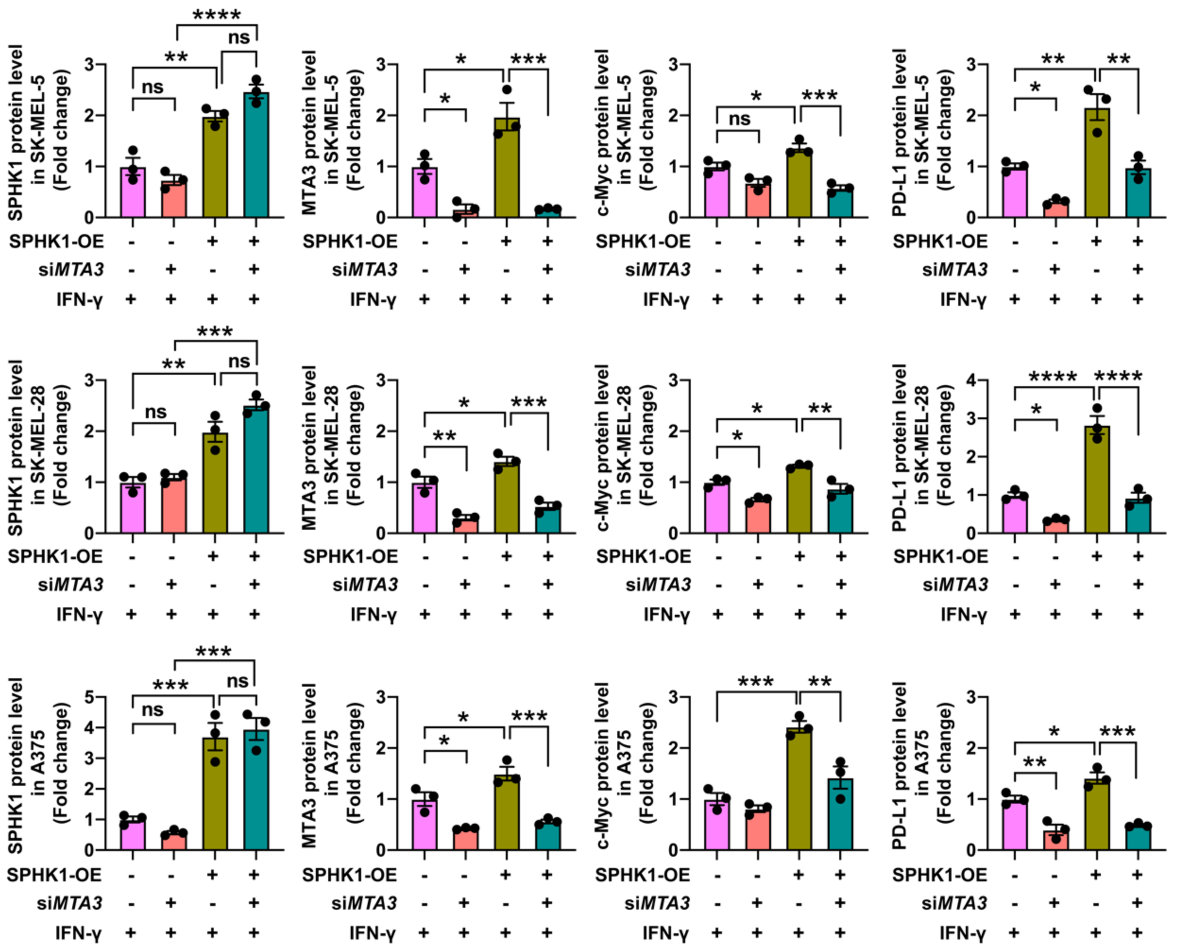

Supplementary Fig. 9, related to Fig. 4.

SK-MEL-5, SK-MEL-28, and A375 cells overexpressing SPHK1 were transfected with si*MTA3* or scrambled negative control si*RNA*. SPHK1, MTA3, c-Myc, or PD-L1 protein levels were analyzed based on western blot. The plot was generated from three independent experiments and showed as mean ± SEM. ns, non-significant, $p > 0.05$, *$p < 0.05$, **$p < 0.01$, ***$p < 0.001$, and ****$p < 0.001$, as determined by Tukey's multiple comparisons test.

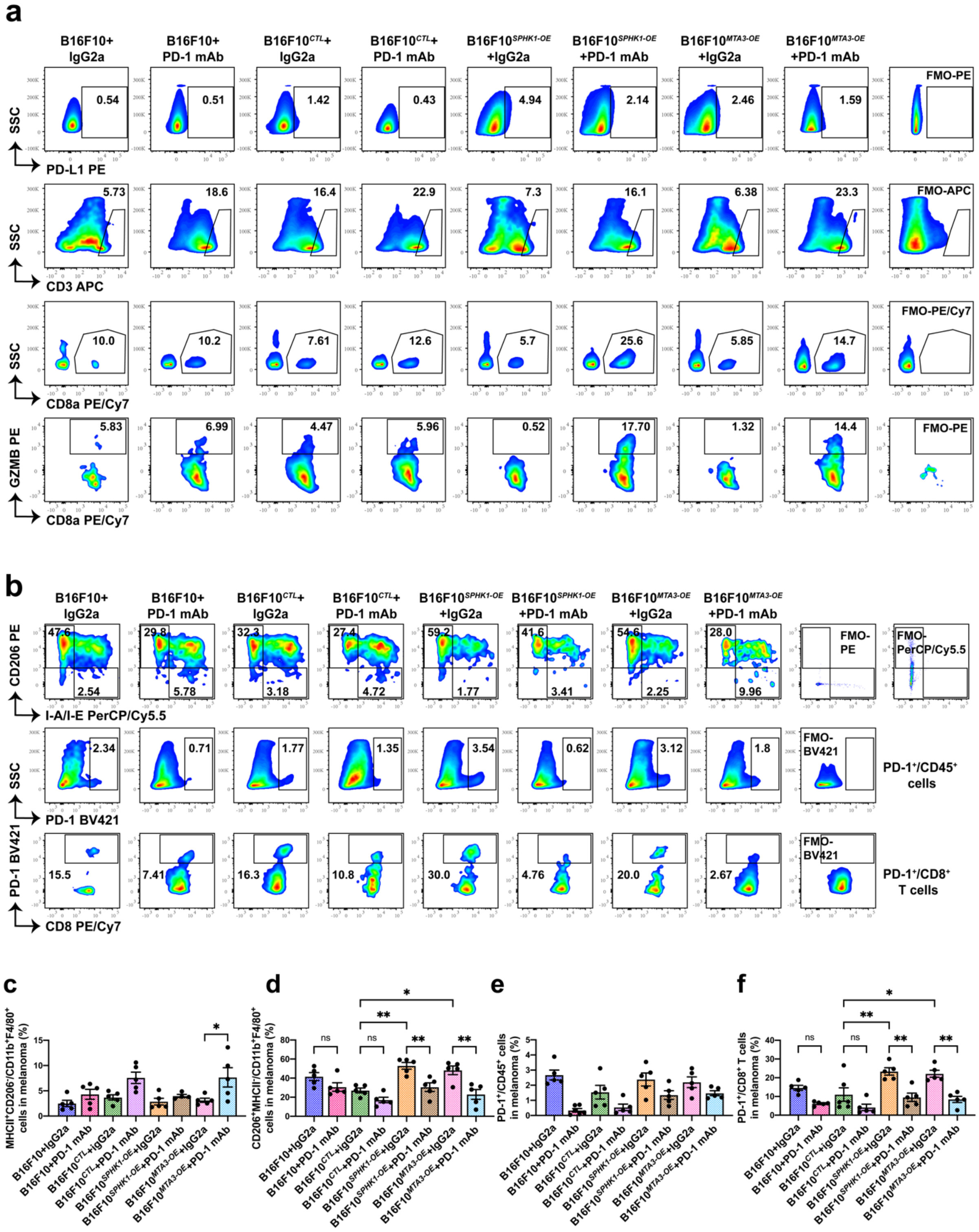
a
B16F10+ IgG2a
B16F10+ PD-1 mAb
B16F10CTL+ IgG2a
B16F10CTL+ PD-1 mAb
B16F10SPHK1-OE +IgG2a
B16F10SPHK1-OE +PD-1 mAb
B16F10MTA3-OE +IgG2a
B16F10MTA3-OE +PD-1 mAb
SSC
PD-L1 PE
0.54
0.51
1.42
0.43
4.94
2.14
2.46
1.59
FMO-PE
CD3 APC
5.73
18.6
16.4
22.9
7.3
16.1
6.38
23.3
FMO-APC
CD8a PE/Cy7
10.0
10.2
7.61
12.6
5.7
25.6
5.85
14.7
FMO-PE/Cy7
GZMB PE
5.83
6.99
4.47
5.96
0.52
17.70
1.32
14.4
FMO-PE
b
CD206 PE
I-A/I-E PerCP/Cy5.5
47.6
2.54
29.8
5.78
32.3
3.18
27.4
4.72
59.2
1.77
41.6
3.41
54.6
2.25
28.0
9.96
FMO-PE
FMO-PerCP/Cy5.5
PD-1 BV421
2.34
0.71
1.77
1.35
3.54
0.62
3.12
1.8
FMO-BV421
PD-1+/CD45+ cells
CD8 PE/Cy7
15.5
7.41
16.3
10.8
30.0
4.76
20.0
2.67
PD-1+/CD8+ T cells
c
MHCII+CD206-/CD11b+F4/80+ cells in melanoma (%)
d
CD206+MHCII-/CD11b+F4/80+ cells in melanoma (%)
e
PD-1+/CD45+ cells in melanoma (%)
f
PD-1+/CD8+ T cells in melanoma (%)
ns
*
**

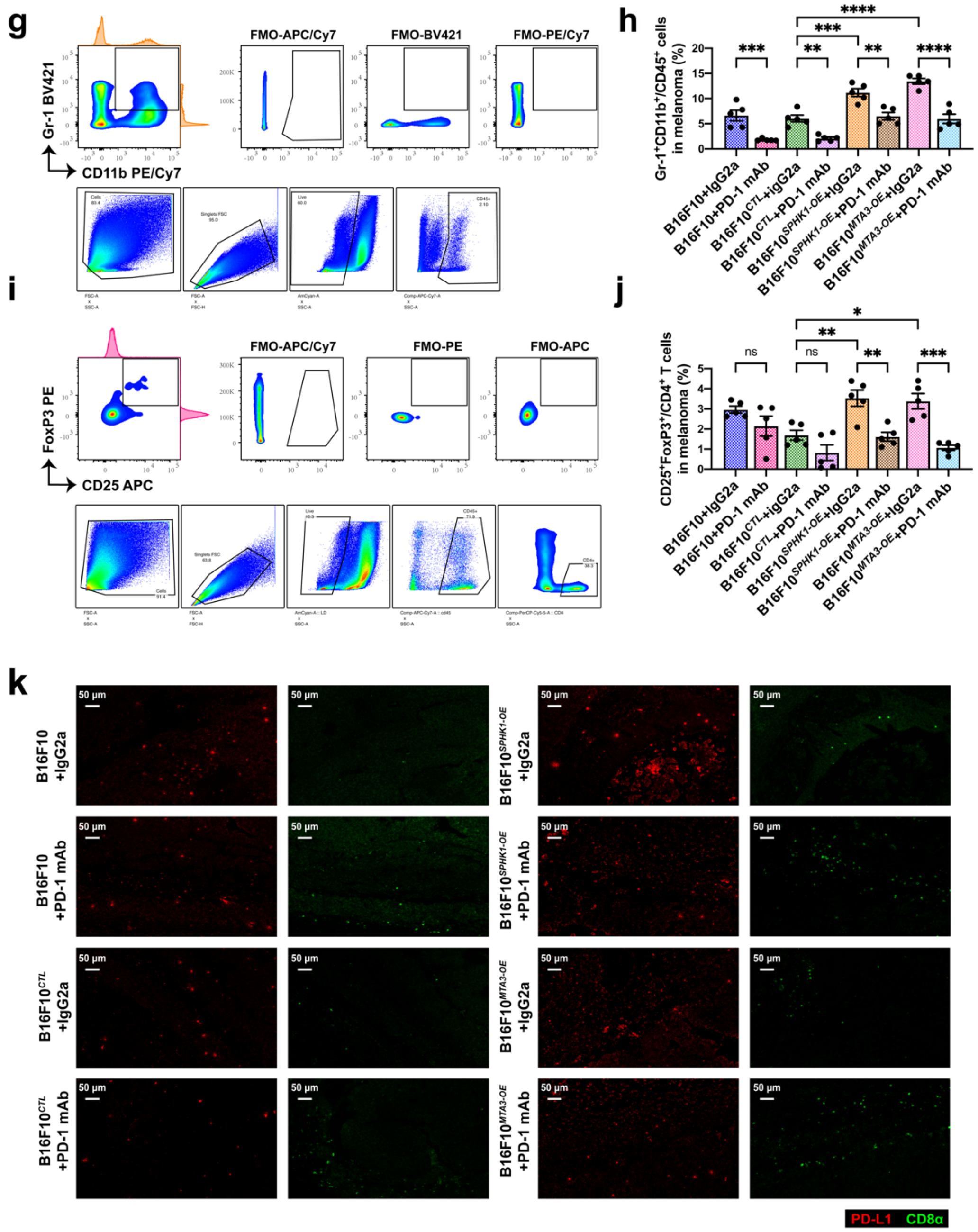

Supplementary Fig. 10, related to Fig. 5.

a, Representative profiles of fluorescence-activated cell sorting of PD-L1 on $CD45^-$ subsets, $CD3^+$ in $CD45^+$ cells, $CD8^+$ in $CD3^+$ cells, and $GZMB^+CD8^+$ TILs from B16F10. **b-f,** Representative profiles and quantification of fluorescence-activated cell sorting of $CD206^-$ MHC class II^+ (**c**) or $CD206^+$MHC class II^- (**d**) in $CD11b^+Gr\text{-}1^+$ cells, PD-1 on $CD45^+$ cells (**e**), and $PD\text{-}1^+CD8^+$ cells (**f**) from B16F10. **g-j,** Gating strategies and quantification of fluorescence-activated cell sorting of $Gr\text{-}1^+CD11b^+$ in $CD45^+$ cells (**g, h**) and $CD25^+FoxP3^+$ in $CD4^+$ cells (**i, j**) from B16F10. **k,** Representative images displaying the individual markers from Fig. 5j. 5 mice per cohort. Data represent mean ± SEM. ns, non-significant, $p > 0.05$, $^*p < 0.05$, $^{**}p < 0.01$, $^{***}p < 0.001$, and $^{****}p < 0.001$, as determined by one-way ANOVA and Tukey's multiple comparisons test (**c-f, h, j**).

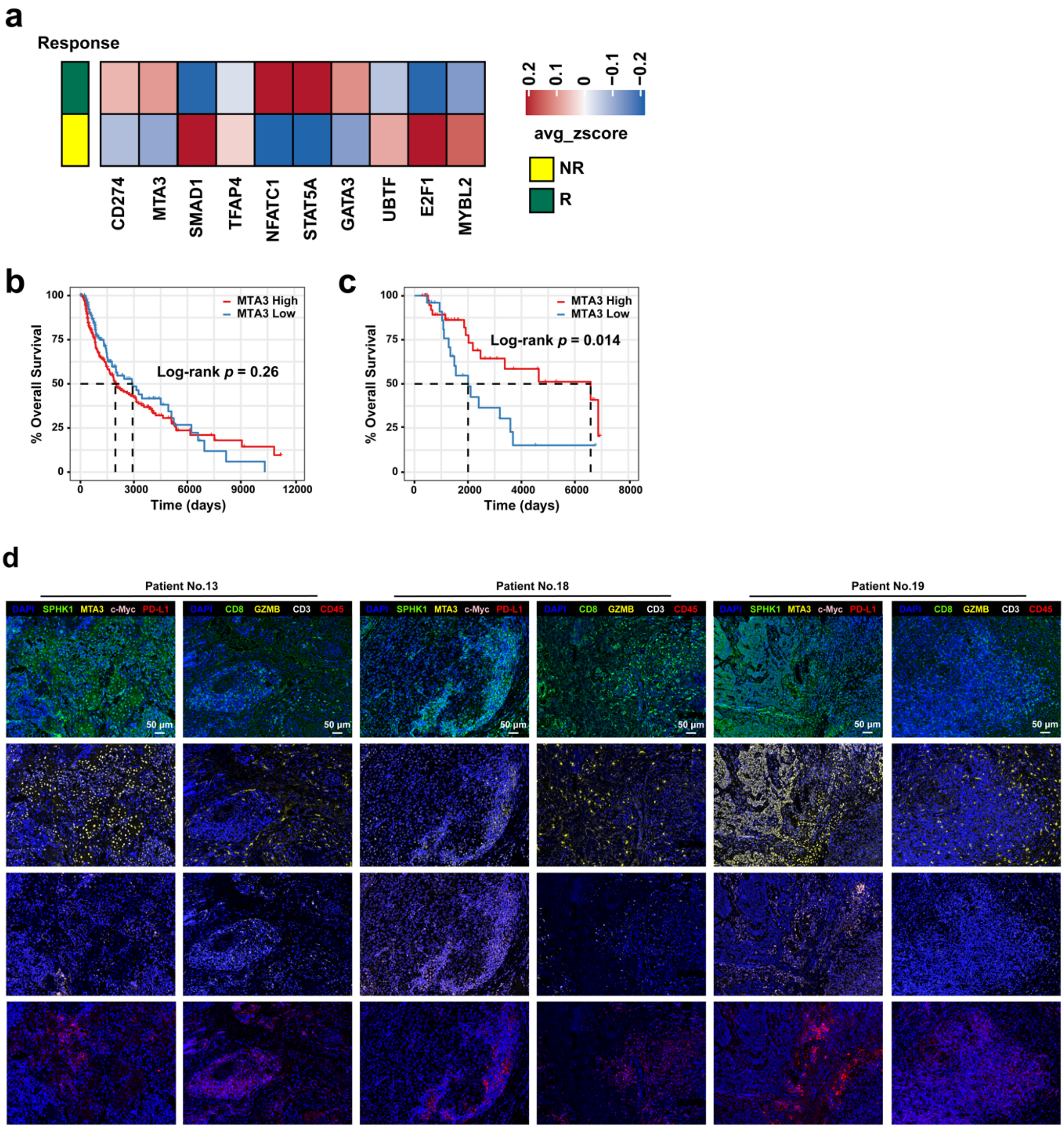
a
Response
CD274
MTA3
SMAD1
TFAP4
NFATC1
STAT5A
GATA3
UBTF
E2F1
MYBL2
0.2
0.1
0
−0.1
−0.2
avg_zscore
NR
R
b
MTA3 High
MTA3 Low
Log-rank p = 0.26
% Overall Survival
Time (days)
c
MTA3 High
MTA3 Low
Log-rank p = 0.014
% Overall Survival
Time (days)
d
Patient No.13
Patient No.18
Patient No.19
DAPI SPHK1 MTA3 c-Myc PD-L1
DAPI CD8 GZMB CD3 CD45
50 μm

e

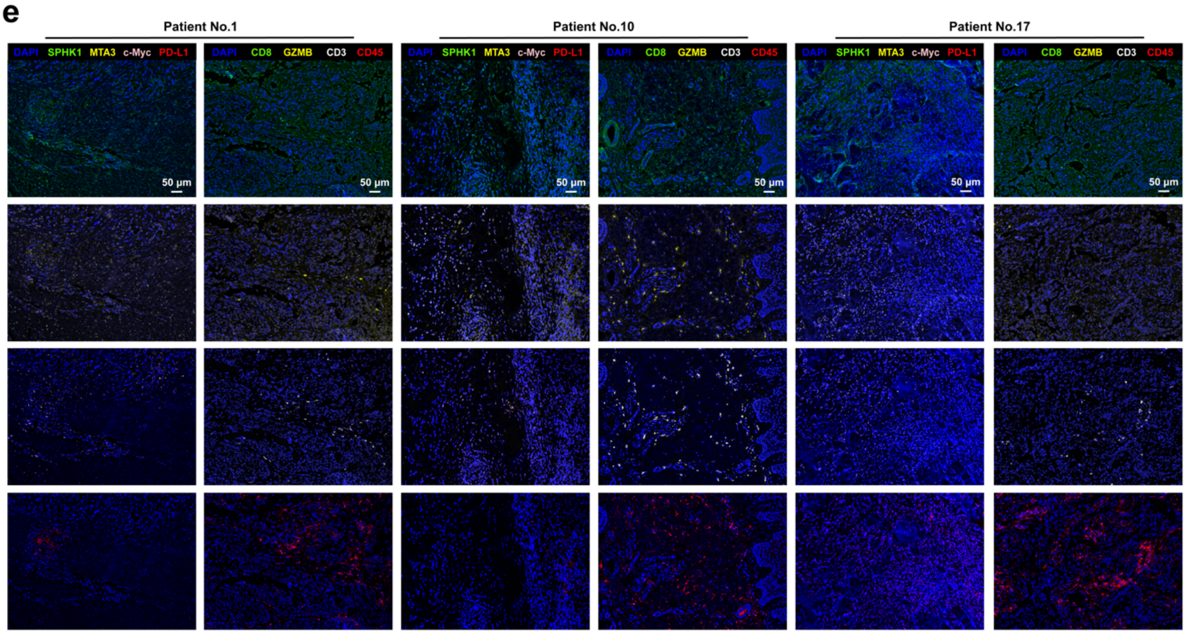

f

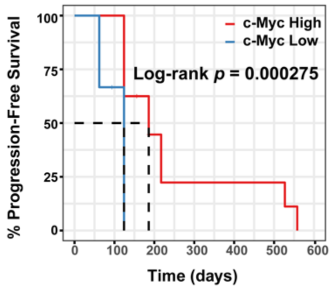

Supplementary Fig. 11, related to Figure 6.

a, Heat map showing the differential gene expression by calculating average z-scores in a total of 28 melanoma patients respond (R, n = 14) or nonrespond (NR, n = 12) to anti-PD-1 therapies. NA, not available (n = 2). **b-c,** Kaplan–Meier estimates for overall survival of SKCM patients from TCGA database. Patients were subdivided according to the treatment (**b**) without or (**c**) with immunotherapies and stratified into two groups: low MTA3 expression (bottom 35%) and high MTA3 expression (the other). Significance was determined by the Log-rank test. **d-e,** Representative images displaying the individual markers together with the DAPI nuclear marker from No.13, No.18, No.19 cases (**d**) and No.1, No.10, No.17 cases (**e**). **f,** Kaplan–Meier survival curves of melanoma patients' progression-free survival. Patients were stratified into two groups by median expression of c-Myc. Significance was determined by the Log-rank test.

Supplementary Table 1, related to Fig.2 Summary of the pairwise comparison *p* values at each time point.

	Days				
	**0**	**2**	**4**	**6**	**8**
**PF543 (5 mg/kg) vs Vehicle**	0.9065	0.6168	0.0516	0.0051	0.0071
**PF543 (10 mg/kg) vs Vehicle**	0.9299	0.4507	0.0091	0.0007	0.0001
**PF543 (10 mg/kg) vs PF543 (5 mg/kg)**	0.7209	0.9584	0.7446	0.3253	0.0050

Supplementary Table 2, related to Fig.2 Summary of a mixed-effect model fitting. The tumor volume data were Log_2-transformed before fitting. The vehicle was set as the reference group. The significant interaction effects between days and treatments suggest that the treatment functions as a higher anti-tumor efficacy as time goes versus the vehicle group.

	**Estimates**	**SEM**	***P***
**Days**	0.49	0.19	0.014
**Body weight**	-0.19	0.26	0.48
**PF543 (5 mg/kg)**	0.27	0.19	0.163
**PF543 (10 mg/kg)**	0.02	0.13	0.87
**Days: PF543 (5 mg/kg)**	1.20	0.19	2.90×10^{-7}
**Days: PF543 (10 mg/kg)**	0.43	0.17	0.019

Supplementary Table 3, related to Supplementary Fig. 3 Summary of the pairwise comparison *p* values at each time point.

	Days				
	**0**	**3**	**6**	**9**	**12**

**Vehicle+CTLA-4 mAb vs Vehicle+IgG2b**	0.9587	0.9061	0.0165	0.0105	0.0002
**PF543+IgG2b vs Vehicle+IgG2b**	0.8686	0.4527	0.0029	0.0606	0.0117
**PF543+CTLA-4 mAb vs Vehicle+IgG2b**	0.9947	0.1181	0.0017	0.0003	<0.0001
**PF543+CTLA-4 mAb vs Vehicle+CTLA-4 mAb**	0.8730	0.3878	0.7127	0.4453	0.0152
**PF543+CTLA-4 mAb vs PF543+IgG2b**	>0.9999	0.9193	0.9749	0.0824	0.0017

Supplementary Table 4, related to Supplementary Fig. 3 Summary of a mixed-effect model fitting. The tumor volume data were Log_2-transformed before fitting. The PF543+CTLA-4 mAb was set as the reference group.

	**Estimates**	**SEM**	***P***
**Days**	0.701	0.348	0.024
**Body weight**	0.902	0.610	0.150
**Vehicle+IgG2b**	0.675	0.664	0.319
**PF543+IgG2b**	0.329	0.644	0.960
**Vehicle+CTLA-4 mAb**	-0.838	0.577	0.157
**Days: Vehicle+IgG2b**	-0.084	0.071	0.245
**Days: PF543+IgG2b**	-0.016	0.069	-0.822
**Days: Vehicle+CTLA-4 mAb**	0.146	0.067	0.038

Supplementary Table 5, related to Fig.3.

Table summary of differentially expressed genes based on RNA sequencing analyses.

Symbol	**Log(FC)**	**Log(CPM)**	**LR**	**FDR**	***P***
MYBL2	-0.80	7.07	260.49	9.70×10^{-57}	1.34×10^{-58}
E2F1	-0.77	4.72	138.33	1.91×10^{-30}	6.17×10^{-32}

UBTF	-0.51	6.69	135.44	7.98×10^{-30}	2.64×10^{-31}
GATA3	-0.50	2.74	20.22	2.89×10^{-5}	6.91×10^{-6}
STAT5A	-0.37	4.68	35.18	2.00×10^{-8}	3.01×10^{-9}
NFATC1	-0.31	3.39	9.85	4.58×10^{-3}	1.70×10^{-3}
MTA3	**-0.30**	**4.58**	**17.23**	$\mathbf{1.24\times10^{-4}}$	$\mathbf{3.30\times10^{-5}}$
SMAD1	-0.28	3.36	8.59	8.52×10^{-3}	3.38×10^{-3}

FC=fold change, CPM=count-per-million, LR=likelihood ratio, FDR=false discovery rate.

Supplementary Table 6, related to Fig.4.

Correlation analyses among PD-L1, c-Myc, SPHK1 and MTA3 expression levels in melanoma patients. Samples were divided into two groups based on the median gene expression. For all of the expected values in cells that were greater than 5, p values were computed using Chi-square tests. Statistical significance was considered with $p < 0.05$.

		**MTA3**			**c-Myc**					**MTA3**		
		**High**	**Low**	**N**	**High**	**Low**	**N**			**High**	**Low**	**N**
	**High**	33	12	45	28	17	45		**High**	35	10	45
**SPHK1**	**Low**	12	33	45	17	28	45	**PD-L1**	**Low**	10	35	45
	**N**	45	45	90	45	45	90		**N**	45	45	90
		Pearson χ^2=19.600, $p < 0.0001$			Pearson χ^2=5.378, $p = 0.020$					Pearson χ^2=27.778, $p < 0.0001$		

Supplementary Table 7, related to Fig.5 Summary of the pairwise comparison p values at each time point.

	**Days**					
	**0**	**2**	**5**	**8**	**11**	**13**
**B16F10+PD-1 mAb vs B16F10+IgG2a**	0.8526	0.9844	>0.9999	0.9860	0.9903	0.9919

**B16F10^{CTL}+PD-1 mAb vs B16F10^{CTL}+IgG2a**	0.9996	>0.9999	0.9924	>0.9999	0.6295	0.1929
**B16F10$^{SPHK1-OE}$+PD-1 mAb vs B16F10$^{SPHK1-OE}$+ IgG2a**	>0.9999	0.9993	0.6802	0.1843	0.0008	<0.0001
**B16F10$^{SPHK1-OE}$+PD-1 mAb vs B16F10^{CTL}+PD-1 mAb**	0.4904	0.8589	0.9964	>0.9999	0.9998	>0.9999
**B16F10$^{SPHK1-OE}$+ IgG2a vs B16F10^{CTL}+IgG2a**	0.8157	0.9867	0.9316	0.3011	0.1716	0.0106
**B16F10$^{MTA3-OE}$+PD-1 mAb vs B16F10$^{MTA3-OE}$+ IgG2a**	0.9984	0.9995	0.9338	0.3801	0.0134	0.0001
**B16F10$^{MTA3-OE}$+PD-1 mAb vs B16F10^{CTL}+PD-1 mAb**	0.7571	0.9746	>0.9999	>0.9999	0.9990	>0.9999
**B16F10$^{MTA3-OE}$+ IgG2a vs B16F10^{CTL}+IgG2a**	>0.9999	0.7315	0.9817	0.7063	0.7516	0.2053

Supplementary Table 8, related to Fig.5 Summary of a mixed-effect model fitting. The tumor volume data were Log_2-transformed before fitting. The SPHK1-OE+PD-1 mAb or MTA3-OE+PD-1 mAb was set as the reference group.

	**Estimates**	**SEM**	***P***		**Estimates**	**SEM**	***P***
**Days**	-0.116	0.571	0.008	**Days**	0.269	0.295	0.009
**Body weight**	0.858	0.105	0.421	**Body weight**	0.247	0.462	0.958
**B16F10+IgG2a**	0.033	0.707	0.963	**B16F10+IgG2a**	0.266	0.334	0.431
**B16F10+PD-1 mAb**	1.041	0.855	0.231	**B16F10+PD-1 mAb**	-0.110	0.404	0.787
**B16F10^{CTL}+IgG2a**	-0.358	0.866	0.682	**B16F10^{CTL}+IgG2a**	-0.322	0.405	0.431
**B16F10^{CTL}+PD-1 mAb**	-0.577	1.47	0.697	**B16F10^{CTL}+PD-1 mAb**	-1.15	0.625	0.074
**B16F10^{SPHK1OE} +IgG2a**	-1.13	0.906	0.220	**B16F10^{MTA3OE} +IgG2a**	0.078	0.406	0.849
**Days: B16F10+IgG2a**	-0.026	0.061	0.669	**Days: B16F10+IgG2a**	-0.110	0.029	0.034
**Days: B16F10 +PD-1 mAb**	0.099	0.080	0.221	**Days: B16F10 +PD-1 mAb**	-0.004	0.038	0.909
**Days:**	0.041	0.083	0.621	**Days:**	0.016	0.039	0.686

**B16F10^{CTL}+IgG2a**				**B16F10^{CTL}+IgG2a**			
**Days: B16F10^{CTL} +PD-1 mAb**	0.050	0.154	0.748	**Days: B16F10^{CTL} +PD-1 mAb**	-0.021	0.021	0.031
**Days: B16F10^{SPHK1OE}+IgG2a**	0.114	0.782	0.153	**Days: B16F10^{MTA3OE}+IgG2a**	-0.001	0.037	0.984

Supplementary Table 9, related to Fig. 6 Clinicopathologic characteristics of PD-1 mAb therapy cohorts.

No.	Age	Sex	TNM Stage	Clinical Stage	Response	PS score	PFS (days)	Metastatic site	Adverse effect
**1**	66	Female	T2aN1M0	IIIA	NR	1	93	Inguinal lymph nodes	None
**2**	53	Female	T4aNxM1c	IV	NR	1	186	Neck, axilla and inguinal lymph nodes, abdominal cavity, peritoneum, lung	None
**3**	38	Female	T3aN2bM0	IIIB	R	1	558^{+}	Axillary, subclavian, and elbow fossa lymph nodes	None
**4**	75	Female	T3bN3M0	IIIC	NR	1	62	Axillary lymph nodes	None
**5**	46	Female	T2bN2cM0	III	NR	1	124	Popliteal lymph nodes	None
**6**	35	Female	TxN2M1	IV	R	1	527	Liver, bone, brain, inguinal lymph nodes	None
**7**	56	Female	T4bN3cM1c	IV	R	1	124^{+}	Inguinal and retroperitoneal lymph nodes	Low cortisol
**8**	56	Female	T1bN1aM0	IIIA	R	1	124^{+}	Inguinal lymph node	Low cortisol
**9**	70	Female	T4bN2bM1a	IV	NR	1	186	Neck and inguinal lymph nodes	None
**10**	71	Male	T3bN3M1a	IV	NR	1	217	Inguinal lymph nodes, left leg skin	None
**11**	46	Male	T4bN0M1	IV	NR	1	155	Lung	None
**12**	37	Female	T1aN1aM0	IIIA	R	1	124^{+}	Popliteal lymph node	White blood cell decreased
**13**	58	Female	T4bN1aM0	IIIB	R	1	124^{+}	Inguinal lymph node	None
**14**	68	Male	T3bN1aM0	IIIA	R	1	124^{+}	Inguinal lymph node	Transient mania, psychosis

**15**	11	Female	T4aN2bM0	IIIB	R	1	124^{+}	Inguinal lymph nodes	None
**16**	68	Female	T3bN2bM1c	IV	R	1	93^{+}	Inguinal lymph nodes, lung	Nausea, vomiting
**17**	64	Female	TxN2M0	IIIA	NR	1	62	Inguinal lymph nodes	None
**18**	64	Female	T4bN1aM0	IIIB	R	1	124^{+}	Inguinal lymph node	Skin ulceration, ACTH increased
**19**	50	Male	T4bN0M0	IIC	R	1	124^{+}	None	None

TNM stage based on the 8th Edition Melanoma Stage Classification. PS, Performance Status; PFS, progression-free survival; OS, overall survival. ACTH=adrenocorticotropic hormone. All patients were treated with PD-1 monoclonal antibody after surgical resection of primary lesions, and those with recurrence or metastasis ⩽ 6 months were considered as nonresponders (NR), while those without recurrence or metastasis longer than 6 months were considered as responders (R).

Supplementary Table 10, related to Fig. 6 Association analyses between PD-L1 and SPHK1 or MTA3 expression levels in melanoma patients. For the expected values in cells that were less than 5, *P* values were computed using Fisher's exact tests. Statistical significance was considered with $p < 0.05$.

		**SPHK1**			**MTA3**		
		**High**	**Low**	**N**	**High**	**Low**	**N**
**PD-L1**	**High**	9	1	10	7	3	10
	**Low**	1	8	9	1	8	9
	**N**	10	9	19	8	11	19
		$p = 9.85\times10^{-4}$			$p = 5.48\times10^{-3}$		
